# Bimetallic Iron–Palladium Catalyst System as a Lewis-Acid for the Synthesis of Novel Pharmacophores Based Indole Scaffold as Anticancer Agents

**DOI:** 10.3390/molecules26082212

**Published:** 2021-04-12

**Authors:** Mohammad Shahidul Islam, M. Ali, Abdullah Mohammed Al-Majid, Abdullah Saleh Alamary, Saeed Alshahrani, Sammer Yousuf, Muhammad Iqbal Choudhary, Assem Barakat

**Affiliations:** 1 Department of Chemistry, College of Science, King Saud University, P.O. Box 2455, Riyadh 11451, Saudi Arabia; mislam@ksu.edu.sa (M.S.I.); maly.c@ksu.edu.sa (M.A.); amajid@ksu.edu.sa (A.M.A.-M.); alamary1401@yahoo.com (A.S.A.); chemistry99y@gmail.com (S.A.); 2 H.E.J. Research Institute of Chemistry, International Center for Chemical and Biological Sciences, University of Karachi, Karachi 75270, Pakistan; dr.sammer.yousuf@gmail.com (S.Y.); iqbal.choudhary@iccs.edu (M.I.C.); 3 Department of Chemistry, Faculty of Science, Alexandria University, P.O. Box 426, Ibrahimia, Alexandria 21321, Egypt

**Keywords:** Friedel–Crafts reaction, Lewis acids, indoles, benzothiophene, benzofuran, anti-cancer activity

## Abstract

The Friedel–Crafts reaction between substituted indoles as nucleophiles with chalcones-based benzofuran and benzothiophene scaffolds was carried out by employing a highly efficient bimetallic iron–palladium catalyst system. This catalytic approach produced the desired *bis*-heteroaryl products with low catalyst loading, a simple procedure, and with acceptable yield. All synthesized indole scaffolds **3a**–**3s** were initially evaluated for their cytotoxic effect against human fibroblast BJ cell lines and appeared to be non-cytotoxic. All non-cytotoxic compounds **3a**–**3s** were then evaluated for their anticancer activities against cervical cancer HeLa, prostate cancer PC3, and breast cancer MCF-7 cell lines, in comparison to standard drug doxorubicin, with IC_50_ values 1.9 ± 0.4 µM, 0.9 ± 0.14 µM and 0.79 ± 0.05 µM, respectively, and appeared to be moderate to weak anticancer agents. Fluoro-substituted chalcone moiety-containing compounds, **3b** appeared to be the most active member of the series against cervical HeLa (IC_50_ = 8.2 ± 0.2 µM) and breast MCF-7 cancer cell line (IC_50_ = 12.3 ± 0.04 µM), whereas 6-fluroindol-4-bromophenyl chalcone-containing compound **3e** (IC_50_ = 7.8 ± 0.4 µM) appeared to be more active against PC3 prostate cancer cell line.

## 1. Introduction

**Figure 1:**
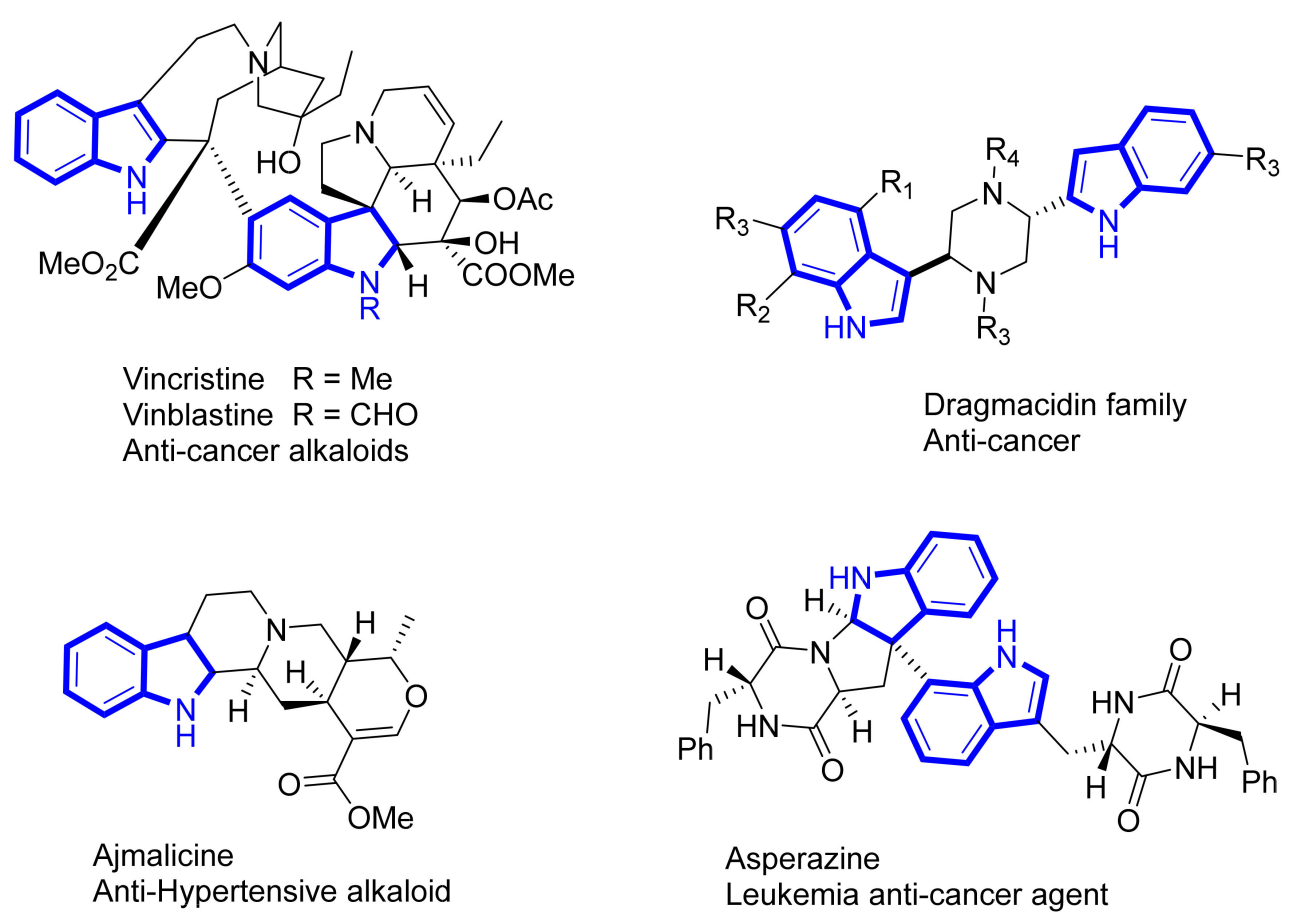
Bioactive compounds incorporating indole skeleton.

Nitrogen heterocycles (natural and synthetic) are key components of several biochemical, and therefore possess interesting biological properties. Indoles are one such class of organic heterocyclic compounds that occur frequently as a privileged structural motif in many synthetic and natural products with versatile pharmacological activities [1]. In many cases they have widespread uses as cholesterol lowering agents, antiviral, antibacterial, antifungal, and as anticancer compounds [2]. Some of them regulate cellular signaling and numerous neuropsychological processes. The indole structures have a rich history in chemistry beginning with the study of dyes in the mid-19th century. Despite the reported toxicity of some indole-containing compounds, several of them possess clinically beneficial properties. The natural products vinblastine and vincristine, isolated from *C. roseus* [3], are active against leukemia, lymphoma, breast, lung and other cancers (Figure 1) [4]. Analogues of these compounds are in use for the treatment of a variety of cancers [5]. Numerous other indole-containing compounds have been shown to possess anti-cancer activity, as well as a myriad of other biological properties [6,7,8]. Because of the prevalence of the indole moiety in biologically active compounds, it is considered a “privileged structure” and that makes it a logical motif to improve biological activity of synthetic compounds [9]. The indole moiety is also found in many natural products and comprises a major subset for the alkaloid class of natural products. For example, ajmalicine is an anti-hypertensive alkaloid [10], asperazine has an unusual profile of cytotoxicity [11,12], and dragmacidin exhibits anti-tumor activity against P-388 cell lines with IC_50_ value of 15 μg/mL, and IC_50_ of 1–10 μg/mL against MDAMB (human mammary), HCT-8 (human colon) and A-549 (human lung) cancer cell lines [13,14,15].

Cancer is one of the leading causes of death globally, with almost 10 million deaths worldwide in 2020 [16]. By 2030, it will become the leading cause of death [17]. 70% of cancer deaths occur in middle or low-resource countries, where as 80% of cases are diagnosed too late for effective treatment. Cancer related deaths are increasing globally [17].

The heterogeneity of tumors, the lack of selectivity of drugs, and the development of drug resistance are some of the major obstacles impeding the effective cure of this deadly disease group. The fact of cell-death makes the situation even worse, because anti-cancer drugs follow first-order kinetics; that is, a fixed percentage, rather than a fixed number of cells are killed by a given treatment. Moreover, individual tumors may contain subpopulations of neoplastic cells that differ in terms of crucial features, any one of which could cause recurrence of the disease [17,18].

Although significant progress has been made in cancer research; still urgent need for a new drug with the advantage of less side effect, safe, overcome drug resistance and high efficacy are challenge to the researcher [19].

In this manuscript, we describe the synthesis of the new hits based on indole/benzofuran/benzothiophene analogues, along with the cytotoxities of the newly synthesized compounds against selected cancer cell lines.

## 2. Results and Discussion

### 2.1. Chemistry

**Scheme 1:**
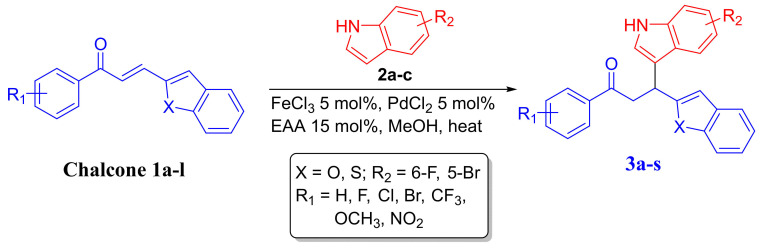
Synthetic routes to **3a**–**3s.** The reaction carried out with optimum reaction conditions as reported by Barakat et al. [23].

**Figure 2:**
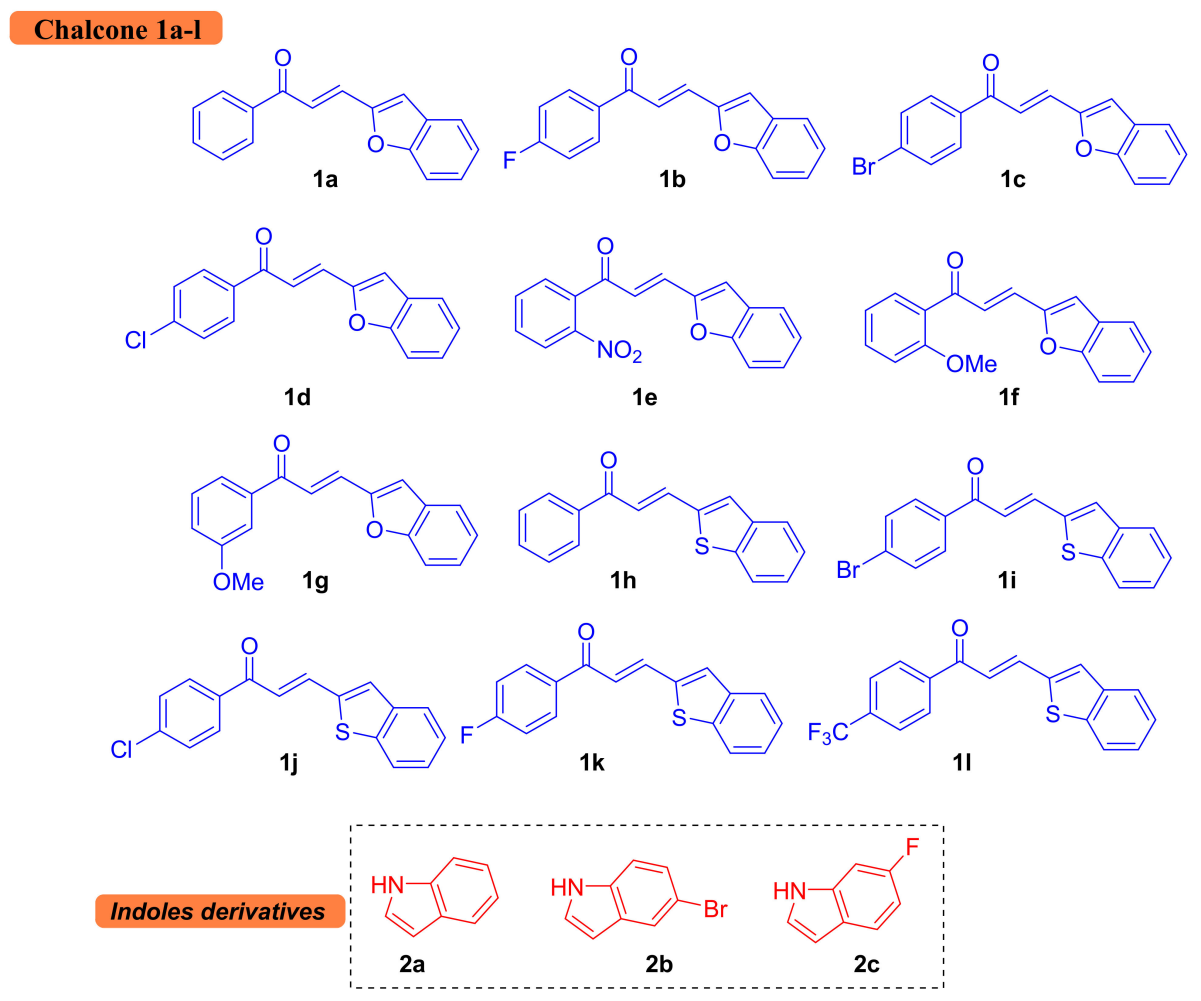
List of synthesized chalcones and substituted indoles utilized in this study.

Michaël-type Friedel–Crafts addition of nucleophiles (especially indoles) with electron-deficient alkenes are fundamentally important chemical transformations [20,21,22,23,24]. These methods have extensive uses in the scientific work on a number of potential nucleophilic species. Stabilized carbanions, organometallic reagents, and heteroatom-based nucleophiles have all been applied to a range of Michael acceptors in previous years [20,21]. The desired chalcones **1a**–**1l** in this study were synthesized by following our previous reported method by Barakat et al [23] (Figure 2). Next, the achiral Friedel–Crafts reaction was explored with the chalcones-based benzofuran or thiophene analogues and the substituted indole by employing Fe-Pd bimetallic catalyst system [23] in MeOH at 60 °C to afford the Friedel–Crafts adducts. To check the productivity and the generality of this approach, the reaction of substituted indoles (indole **2a**; 5-bromoindole **2b**; 6-fluroroindole **2c**) and chalcones based benzofuran or thiophene analogues was carried out under the reaction conditions (10 mol% of FeCl_3_, 10 mol% of PdCl_2_ and 15 mol% ethyl acetoacetate (EAA), 1.0 equiv. chalcone and 1.1 equiv. substituted indole in CH_3_OH at 60 °C; Scheme 1). A range of heteroaryl enone substituents further demonstrated a broad substrate scope. Both electron-deficient and electron-rich heterocycles afforded the Friedel–Crafts products in high yield.

The structures of requisite Friedel–Crafts adducts were determined by using spectroscopic data, including ^1^H-, ^13^C-NMR, Mass, and FT-IR spectroscopy.

### 2.2. Anti-Cancer Activity

All synthesized indoles **3a**–**3s** were first evaluated for their cytotoxic effect against human fibroblast BJ cell line, and appeared to be either non-toxic (**3a**, **3i**–**3k, 3r** and **3s**) or slightly toxic than (30%) **3b**–**3i,** and **3f**–**3q** at a concentration of 30 µM. Based on the largely non-toxic nature of newly synthesized compounds against human fibroblast cell line compounds **3a**–**3s** were further evaluated for their anticancer activities against HeLa, PC3, and MCF-7 cancer cell lines in comparison to standard drug doxorubicin, with IC_50_ values 1.9 ± 0.4 µM, 0.9 ± 0.14 µM and 0.79 ± 0.05 µM, respectively, and appeared to be moderate to weak anticancer agents. Results of cytotoxic analysis and anti-cancer activities are summarized in Table 1.

Among series of substituted indoles and chalcone-based benzofurans compounds, **3a**–**3m** and **3r**–**3s**, 6-fluoroindol-4-bromophenyl chalcone-containing compound **3e** (IC_50_ = 7.8 ± 0.4 µM) appeared to be the most active anti-cancer agent against prostate PC3 cancer cell line in comparison to non-substituted indoles and chalcone-based benzofuran (**3a**, IC_50_ = 11.4 ± 0.2 µM) and drug doxorubicin (IC_50_ = 1.9 ± 0.4 µM). However, decrease in activity was observed in compounds **3b** (IC_50_ = 22.2 ± 0.4 µM), **3d** (IC_50_ = 26.3 ± 0.8 µM), and **3f** (IC_50_ = 27.0 ± 0.4 µM) having non-fluorinated indole moieties with fluoro-, bromo-, and chloro-substituted chalcone moieties, respectively. Similar pattern of decrease of activity was observed in compounds **3g** (IC_50_ = 20.0 ± 1.3 µM), having chloro substituted phenyl rings of chalcones instead of bromo-substituted chalcone moiety of containing compound **3e** (IC_50_ = 7.8 ± 0.4 µM). A complete loss of anticancer activity against PC3 cells was observed when fluoro-substituted chalcone moieties were replaced by *o*-nitro phenyl ring of containing **3i**. Anti-cancer potential was restored in indole and chalcone-based benzofuran derivative **3j** (IC_50_ = 16.0 ± 0.1 µM) with bromo-substituted indole ring instead of fluorine substituent **3i**. Among series of compounds having methoxy-substituted chalcone moiety **3m** (IC_50_ = 19.3 ± 0.1 µM) appeared as weak anti-cancer agents whereas compounds **3r** and **3s** appeared to be inactive. The pattern of activity of compound **3f** (IC_50_ = 16.3 ± 1.6 µM) and **3r** (in active) clearly demonstrated the positional effect of methoxy substituent of the chalcone moiety towards anti-cancer activity. All members of the series of the indole and chalcone-based thiophene derivatives **3n**–**3q**, appeared as weak anti-cancer agents against prostate cancer PC3 cell line in comparison to doxorubicin (IC_50_ = 1.9 ± 0.4 µM) as standard drug.

All synthesized substituted indole and chalcone-based benzofurans **3a**–**3m** and **3r**–**3s** were further evaluated for their anticancer activities against cervical cancer HeLa cell lines and appeared to be moderate to weak anticancer agents against standard doxorubicin (IC_50_ = 0.9 ± 0.14 µM). Non-substituted indole and chalcone-based benzofurans **3a** appeared as weak anti-cancer agent with IC_50_ = 22.5 ± 0.9 µM. However, substitution of fluorine on phenyl ring of chalcone moiety contributed towards drastic increase in anti-cancer potential as observed in **3b** (IC_50_ = 8.2 ± 0.2 µM), followed by gradual decrease in anti-cancer potential by various substituents on chalcone and indole moieties as observed in 6-bromoindol-4-flurophenyl chalcone-containing **3c** (IC_50_ = 10.8 ± 0.1 µM), 4-bromophenyl chalcone-containing **3d** (IC_50_ = 12.6 ± 0.4 µM), 6-fluroindol-4-bromophenyl chalcone-containing **3e** (IC_50_ = 13.6 ± 0.4 µM), 4-chlorophenyl chalcone-containing **3f** (IC_50_ = 11.9 ± 0.2 µM), 6-fluroindol-4-chlorophenyl chalcone-containing **3g** (IC_50_ = 14.7 ± 1.1 µM), 4-aminophenyl chalcone-containing **3h** (IC_50_ = 29.7 ± 0.4 µM), 6-fluroindol-2-nitrophenyl chalcone-containing **3i** (IC_50_ = 15.2 ± 0.6 µM), 5-bromoroindol-2-nitrophenyl chalcone-containing **3j** (IC_50_ = 17.3 ± 0.7 µM), 4-trifluoromethylphenyl chalcone-containing **3k** (IC_50_ = 14.9 ± 0.6 µM), 3-methoxyphenyl chalcone-containing **3l** (IC_50_ = 11.1 ± 0.6 µM), 6-fluoroindol-3-methoxyphenyl chalcone-containing **3m** (IC_50_ = 15.2 ± 0.2 µM), and 5-bromoindol-2-methoxyphenyl chalcone-containing **3s** (IC_50_ = 18.4 ± 0.2 µM). Similar activity pattern was observed in non-substituted indole and chalcone-based thiophene derivative **3n** (IC_50_ = 12.4 ± 0.2 µM), 4-chlorophenyl chalcone-containing compound **3o** (IC_50_ = 13.8 ± 0.3 µM), 4-fluorophenyl chalcone-containing **3p** (IC_50_ = 12.7 ± 0.1 µM), and 4-trifluoromethylphenyl chalcone-containing **3q** (IC_50_ = 18.8 ± 0.2 µM against cervical cancer cell line HeLa in comparison to doxorubicin (IC_50_ = 1.9 ± 0.4 µM) as standard drug.

Many of the tested compounds appeared to be inactive against MCF-7 breast cancer cell lines, except **3b, 3c, 3f, 3m** and **3s**. Non-substituted indole and chalcone-based benzofurans **3a**, appeared to be inactive against MCF-7 breast cancer cell lines; however, substitution of fluorine on phenyl ring of chalcone moiety contributed towards drastic increase in anti-cancer potential, as observed in **3b** (IC_50_ = 12.37 ± 0.04 µM), followed by gradual decrease in anti-cancer potential due to various substituents on chalcone and indole moieties, as observed in 4-chlorophenyl chalcone-containing **3f** (IC_50_ = 19.28 ± 0.78 µM), 6-fluroindol-3-methoxyphenyl chalcone-containing **3m** (IC_50_ = 22.9 ± 0.43 µM), 6-bromoindol-4-flurophenyl chalcone-containing **3c** (IC_50_ = 25.87 ± 0.8 µM), and 5-bromoindol-2-methoxyphenyl chalcone-containing compound **3s** (IC_50_ = 28.64 ± 0.3 µM) against standard doxorubicin (IC_50_ = 0.79 ± 0.4 µM). All synthesized indole and chalcone-based thiophene derivatives **3n**–**3q** appeared to be inactive against breast cancer cell line MCF-7.

The comparison of substituted indole and chalcone-based benzofurans (**3a**, **3b**, **3g**, and **3k**) with substituted indole and chalcone-based thiophenes (**3r**–**3s**) disclosed that non-substituted indole and chalcone-based benzofuran **3a** (IC_50_ = 11.4 ± 0.2 µM) and fluoro-, and chloro-substituted chalcone moieties, containing compounds **3b** (IC_50_ = 22.2 ± 0.5 µM) and **3g** (IC_50_ = 22.0 ± 1.3 µM), respectively, appeared to be more active against PC3 prostate cancer cell lines, in comparison to non-substituted indole and chalcone-based thiophene **3r** (IC_50_ = 20.4 ± 0.4 µM) and fluoro-, and chloro-substituted chalcone moieties, containing compounds **3p** (IC_50_ = 27.6 ± 0.5 µM) and **3s** (IC_50_ = 29.7 ± 0.5 µM), respectively, that appeared to have more anti-cancer potential against cervical cancer HeLa cell line **3r** (IC_50_ = 12.4 ± 0.2 µM), **3o** (IC_50_ = 13.8 ± 0.3 µM) and **3p** (IC_50_ = 12.7 ± 0.1 µM)]. Among fluoro-, and chloro-substituted chalcone-containing compounds **3b** and **3s,** respectively. Whereas trifluoro-substituted chalcone-containing benzofuran **3k** (IC_50_ = 14.9 ± 0.6 µM) showed more activity against cervical cancer HeLa cell line, in comparison to thiophene analogue (**3q**, IC_50_ = 18.8 ± 0.2 µM). Among benzofurans (**3a**, **3b**, **3g**, and **3k**) and thiophene **3r**–**3s** structural analogues, 4-fluorophenyl chalcone-containing **3b** (IC_50_ = 12.37 ± 0.04 µM) appeared to be the only active member against MCF-7 breast cancer cell line. All results are summarized in Table 1.

## 3. Materials and Methods

### 3.1. General Procedure (GP1)

Chalcones **1a–1l** (0.5 mmol), indoles (0.55 mmol), and dry methanol (20 mL) were charged into a 100 mL round bottom flask, equipped with condenser under inert atmosphere. FeCl_3_ (5 mol%), PdCl_2_ (5 mol%), and 15 mol% of ethyl-acetoacetate (EAA) were added to the reaction mixture. Then the reaction was heated at 60–80 °C for 2–3 h. The reaction was monitored by TLC until the complete consumption of starting materials. Then the reaction mixture was allowed to cool and 20 mL of distilled water was added. The products were extracted in CH_2_Cl_2_ (3 × 20 mL) and the organics part were dried over anhydrous Mg_2_SO_4_. The organic phases were concentrated under reduced pressure and subjected to column chromatography using 100–200 mesh silica gel and 20–30% Ethyl acetate in *n*-hexane to afford moderate to good yields (60–90%).

### 3.2. Synthesis of 3-(Benzofuran-2-yl)-3-(1H-indol-3-yl)-1-phenylpropan-1-one (***3a***)

Following the general procedure (GP1) chalcone **1a** (124 mg, 0.5 mmol) and indole **2a** (64 mg, 0.55 mmol) produces Friedel–Crafts product-**3a** (yield 159 mg, 87%); m.p. 114–115 °C; ^1^H-NMR (500 MHz, DMSO-*d*_6_) δ 10.97 (d, *J* = 2.4 Hz, 1H, NH), 8.08–8.02 (m, 2H, Ar-H), 7.66–7.60 (m, 2H, Ar-H), 7.55–7.48 (m, 3H, Ar-H), 7.44–7.40 (m, 1H, Ar-H), 7.38–7.32 (m, 2H, Ar-H), 7.16 (pd, *J* = 7.3, 1.5 Hz, 2H, Ar-H), 7.06 (ddd, *J* = 8.1, 7.0, 1.2 Hz, 1H, Ar-H), 6.96 (ddd, *J* = 7.9, 6.9, 1.0 Hz, 1H, Ar-H), 6.70 (d, *J* = 1.0 Hz, 1H, Ar-H), 5.12 (t, *J* = 7.2 Hz, 1H, CH), 4.07 (dd, *J* = 17.5, 7.6 Hz, 1H, CH_2(a)_), 3.93 (dd, *J* = 17.5, 7.0 Hz, 1H, CH_2(b)_); ^13^C-NMR (126 MHz, DMSO-*d*_6_) δ 197.7 (CO), 161.0, 153.9, 136.6, 136.3, 133.3, 128.7, 128.4, 128.1, 126.1, 123.3, 122.9, 122.6, 121.1, 120.5, 118.7, 118.6, 114.3, 111.6, 110.7, 101.8, 41.9 (CH), 31.8 (CH_2_); IR (KBr, cm^−1^) ν_max_ = 3404, 3056, 29.04,2848, 1678, 1589, 1450, 1415, 1334, 1253; [Anal. Calcd. for C_25_H_19_NO_2_: C, 82.17; H, 5.24; N, 3.83; Found: C, 82.29; H, 5.12; N, 3.65]; LC/MS (ESI, *m*/*z*): 366.15 [M + H]^+^, exact mass 365.14 for C_25_H_19_NO_2_.

### 3.3. Synthesis of 3-(Benzofuran-2-yl)-1-(4-fluorophenyl)-3-(1H-indol-3-yl)propan-1-one (***3b***)

Following the general procedure (GP1) chalcone **1b** (133 mg, 0.5 mmol) indole **2a** (64 mg, 0.55 mmol) produces Friedel–Crafts product-**3b** (yield 174 mg, 91%); m.p. 157–158 ^o^C; ^1^H-NMR (500 MHz, DMSO-*d*_6_) δ 10.98 (d, *J* = 2.4 Hz, 1H, NH), 8.16−8.10 (m, 2H, Ar-H), 7.64 (d, *J* = 7.9 Hz, 1H, Ar-H), 7.50 (dd, *J* = 6.8, 2.2 Hz, 1H, Ar-H), 7.44−7.40 (m, 1H, Ar-H), 7.38–7.32 (m, 4H, Ar-H), 7.16 (td, *J* = 7.0, 1.6 Hz, 2H, Ar-H), 7.06 (ddd, *J* = 8.1, 6.9, 1.2 Hz, 1H, Ar-H), 6.98–6.93 (m, 1H, Ar-H), 6.70 (d, *J* = 1.0 Hz, 1H, Ar-H), 5.11 (t, *J* = 7.2 Hz, 1H, CH), 4.07 (dd, *J* = 17.5, 7.5 Hz, 1H, CH_2(a)_), 3.92 (dd, *J* = 17.5, 6.9 Hz, 1H, CH_2(b)_); ^13^C-NMR (126 MHz, DMSO-*d*_6_) δ 196.9 (CO), 166.6 & 164.6 (C_1_-F, *J*_C-F_ = 200.3 Hz), 161.5, 154.4, 136.9, 133.9, 133.9, 131.7 & 131.6 (C_4_-F, *J*_C-F_ = 7.4 Hz), 128.9, 126.6, 123.9, 123.5, 123.10, 121.6, 121.1, 119.2 &119.1 (C_3_-F, *J*_C-F_ = 11.1 Hz), 116.3 & 116.2 (C_2_-F, *J*_C-F_ = 17.4 Hz), 114.8, 112.1, 111.3, 102.4, 42.4 (CH), 32.3(CH_2_); IR (KBr, cm^−1^) ν_max_ = 3331, 3055, 2904, 1677, 1591, 1502, 1452, 1363, 1340, 1234, 1225; [Anal. Calcd. for C_25_H_18_FNO_2_: C, 78.31; H, 4.73; N, 3.65; Found: C, 78.19; H, 4.65; N, 3.52]; LC/MS (ESI, *m*/*z*): 384.14 [M + H]^+^, exact mass 383.13 for C_25_H_18_FNO_2_.

### 3.4. Synthesis of 3-(Benzofuran-2-yl)-3-(5-bromo-1H-indol-3-yl)-1-(4-fluorophenyl)propan-1-one (***3c***)

The general procedure (GP1) chalcone **1b** (133 mg, 0.5 mmol) 5-bromoindole **2b** (108 mg, 0.55 mmol) produces Friedel–Crafts product-**3c** (yield 191 mg, 83%); m.p. 167–168 °C; ^1^H-NMR (400 MHz, DMSO-*d*_6_) δ 11.21 (d, *J* = 2.4 Hz, 1H, NH), 8.13 (dd, *J* = 8.7, 5.8 Hz, 2H, Ar-H), 7.82 (d, *J* = 2.0 Hz, 1H, Ar-H), 7.54–7.50 (m, 1H, Ar-H), 7.47–7.41 (m, 2H, Ar-H), 7.34 (t, *J* = 8.9 Hz, 3H, Ar-H), 7.22–7.12 (m, 3H, Ar-H), 6.73 (s, 1H, Ar-H), 5.10 (t, *J* = 7.1 Hz, 1H, CH), 4.06 (dd, *J* = 17.7, 7.3 Hz, 1H, CH_2(a)_), 3.93 (dd, *J* = 17.7, 7.2 Hz, 1H, CH_2(b)_); ^13^C-NMR (101 MHz, DMSO-*d*_6_) δ 196.2 (CO), 166.4 & 163.9 (C_1_-F, *J*_C-F_ = 250.7 Hz), 160.6, 153.9, 134.9, 133.3, 131.2 & 131.1 (C_4_-F, *J*_C-F_ = 9.6 Hz), 128.4, 127.9, 124.8, 123.6, 123.4, 122.6, 120.9, 120.6, 115.8 & 115.6 (C_2_-F, *J*_C-F_ = 22.2 Hz), 114.2, 113.6, 111.3, 110.7, 101.9, 41.9 (CH), 31.4 (CH_2_); IR (KBr, cm^−1^) ν_max_ = 3396, 3066, 2921, 1675, 1591, 1504, 1452, 1413, 1226; [Anal. Calcd. for C_25_H_17_BrFNO_2_: C, 64.95; H, 3.71; N, 3.03; Found: C, 65.17; H, 3.62; N, 2.93]; LC/MS (ESI, *m*/*z*): 462.05 [M + H] ^+^, exact mass 461.04 for C_25_H_17_BrFNO_2_.

### 3.5. Synthesis of 3-(Benzofuran-2-yl)-1-(4-bromophenyl)-3-(1H-indol-3-yl)propan-1-one (***3d***)

Following the general procedure (GP1) chalcone **1c** (164 mg, 0.5 mmol) indole **2a** (64 mg, 0.55 mmol) produces Friedel–Crafts product-**3d** (yield 189 mg, 85%); m.p. 108–109 °C; ^1^H-NMR (500 MHz, DMSO-*d*_6_) δ 10.99–10.93 (m, 1H, NH), 8.00–7.94 (m, 2H, Ar-H), 7.73 (d, *J* = 8.5 Hz, 2H, Ar-H), 7.62 (d, *J* = 8.0 Hz, 1H, Ar-H), 7.51–7.47 (m, 1H, Ar-H), 7.41 (d, *J* = 7.6 Hz, 1H, Ar-H), 7.37–7.31 (m, 2H, Ar-H), 7.16 (td, *J* = 7.1, 1.7 Hz, 2H, Ar-H), 7.05 (t, *J* = 7.6 Hz, 1H, Ar-H), 6.95 (t, *J* = 7.5 Hz, 1H, Ar-H), 6.70 (s, 1H, Ar-H), 5.08 (t, *J* = 7.3 Hz, 1H, CH), 4.06 (dd, *J* = 17.5, 7.5 Hz, 1H, CH_2(a)_), 3.91 (dd, *J* = 17.5, 6.8 Hz, 1H CH_2(b)_); ^13^C-NMR (126 MHz, DMSO-*d*_6_) δ 197.0 (CO), 160.9, 153.9, 136.3, 135.5, 131.8, 130.2, 128.4, 127.4, 126.1, 123.4, 123.0, 122.6, 121.1, 120.5, 118.7, 118.6, 114.2, 111.6, 110.7, 101.9, 41.9 (CH), 31.7 (CH_2_); IR (KBr, cm^−1^) ν_max_ = 3323, 3057, 2920, 2852, 1678, 1580, 1452, 1394, 1363, 1338, 1252, 1197; [Anal. Calcd. for C_25_H_18_BrNO_2_: C, 67.58; H, 4.08; N, 3.15; Found: C, 67.45; H, 4.19; N, 3.01]; LC/MS (ESI, *m*/*z*): 444.06 [M + H] ^+^, exact mass 443.05 for C_25_H_18_BrNO_2_.

### 3.6. Synthesis of 3-(Benzofuran-2-yl)-1-(4-bromophenyl)-3-(6-fluoro-1H-indol-3-yl)propan-1-one (***3e***)

Following the general procedure (GP1) chalcone **1c** (164 mg, 0.5 mmol) 6-fluoroindole **2c** (74 mg, 0.55 mmol) produces Friedel–Crafts product-**3e** (yield 166 mg, 72%); m.p. 123–124 °C; ^1^H-NMR (400 MHz, DMSO-*d*_6_) δ 11.05 (d, *J* = 2.5 Hz, 1H, NH), 7.97 (d, *J* = 8.1 Hz, 2H, Ar-H), 7.72 (d, *J* = 8.1 Hz, 2H, Ar-H), 7.62 (dd, *J* = 8.8, 5.3 Hz, 1H, Ar-H), 7.50 (d, *J* = 7.1 Hz, 1H, Ar-H), 7.41 (d, *J* = 7.5 Hz, 1H, Ar-H), 7.37 (d, *J* = 2.6 Hz, 1H, Ar-H), 7.22–7.08 (m, 3H, Ar-H), 6.82 (td, *J* = 9.4, 2.5 Hz, 1H, Ar-H), 6.71 (s, 1H, Ar-H), 5.08 (t, *J* = 7.2 Hz, 1H, CH), 4.06 (dd, *J* = 17.6, 7.4 Hz, 1H, CH_2(a)_), 3.90 (dd, *J* = 17.6, 6.8 Hz, 1H, CH_2(b)_); ^13^C-NMR (126 MHz, DMSO-*d*_6_) δ 196.9 (CO), 160.7, 159.8 & 157.9 (C_1_-F, *J*_C-F_ = 186.3 Hz), 153.9, 136.2 & 136.1 (C_3_-F, *J*_C-F_ = 10.3 Hz), 135.5, 131.8, 130.2, 128.4, 127.5, 123.6 & 123.6 (C_4_-F, *J*_C-F_ = 2.6 Hz), 123.4, 122.9, 122.6, 120.6, 119.7 & 119.7 (C_5_-F, *J*_C-F_ = 8.2 Hz), 114.5, 110.7, 107.2 & 106.9 (C_2_-F, *J*_C-F_ = 19.5 Hz), 101.9, 97.6 & 97.4 (C_6_-F, *J*_C-F_ = 20.2 Hz), 41.9 (CH), 31.6 (CH_2_); IR (KBr, cm^−1^) ν_max_ = 3359, 2918, 2850, 1678, 1624, 1581, 1454, 1394, 1336, 1250; [Anal. Calcd. for C_25_H_17_BrFNO_2_: C, 64.95; H, 3.71; N, 3.03; Found: C, 64.84; H, 3.66; N, 3.21]; LC/MS (ESI, *m*/*z*): 462.05 [M + H], exact mass 461.04 for C_25_H_17_BrFNO_2_.

### 3.7. Synthesis of 3-(Benzofuran-2-yl)-1-(4-chlorophenyl)-3-(1H-indol-3-yl)propan-1-one (***3f***)

Following the general procedure (GP1) chalcone **1d** (141 mg, 0.5 mmol) indole **2a** (64 mg, 0.55 mmol) produces Friedel–Crafts product-**3f** (yield 158 mg, 79%); m.p. 126–127 °C; ^1^H-NMR (400 MHz, DMSO-*d*_6_) δ 10.98 (d, *J* = 2.4 Hz, 1H, NH), 8.05 (d, *J* = 8.2 Hz, 2H, Ar-H), 7.63 (d, *J* = 8.0 Hz, 1H, Ar-H), 7.58 (d, *J* = 8.6 Hz, 2H, Ar-H), 7.52–7.47 (m, 1H, Ar-H), 7.44–7.40 (m, 1H, Ar-H), 7.38–7.33 (m, 2H, Ar-H), 7.16 (t, *J* = 5.6 Hz, 2H, Ar-H), 7.06 (t, *J* = 7.7 Hz, 1H, Ar-H), 6.96 (t, *J* = 7.4 Hz, 1H, Ar-H), 6.70 (s, 1H, Ar-H), 5.10 (t, *J* = 7.2 Hz, 1H, CH), 4.07 (dd, *J* = 17.6, 7.4 Hz, 1H, CH_2(a)_), 3.93 (dd, *J* = 17.6, 7.1 Hz, 1H, CH_2(b)_); ^13^C-NMR (101 MHz, DMSO-*d*_6_) δ 197.4 (CO), 161.5, 154.5, 138.8, 136.9, 135.8, 130.6, 129.4, 128.9, 126.7, 123.9, 123.6, 123.2, 121.7, 121.1, 119.3, 119.1, 114.8, 112.1, 111.3, 102.5, 42.6 (CH), 32.3 (CH_2_); IR (KBr, cm^−1^) ν_max_ = 3323, 2935, 2918, 2854, 1680, 1585, 1456, 1396, 1362, 1340, 1250; [Anal. Calcd. for C_25_H_18_ClNO_2_: C, 75.09; H, 4.54; N, 3.50; Found: C, 75.23; H, 4.46; N, 3.39]; LC/MS (ESI, *m*/*z*): 400.14 [M + H] ^+^, exact mass 399.10 for C_25_H_18_ClNO_2_.

### 3.8. Synthesis of 3-(Benzofuran-2-yl)-1-(4-chlorophenyl)-3-(6-fluoro-1H-indol-3-yl)propan-1-one (***3g***)

Following the general procedure (GP1) chalcone **1d** (141 mg, 0.5 mmol) 6-fluoroindole **2c** (74 mg, 0.55 mmol) produces Friedel–Crafts product-**3g** (yield 146 mg, 70%); m.p. 112–113 °C; ^1^H-NMR (400 MHz, DMSO-*d*_6_) δ 11.08 (d, *J* = 2.4 Hz, 1H, NH), 8.05 (d, *J* = 8.6 Hz, 2H, Ar-H), 7.64 (dd, *J* = 8.8, 5.6 Hz, 1H, Ar-H), 7.57 (d, *J* = 8.3 Hz, 2H, Ar-H), 7.50 (d, *J* = 7.4 Hz, 1H, Ar-H), 7.44–7.36 (m, 2H, Ar-H), 7.21–7.10 (m, 3H, Ar-H), 6.84 (td, *J* = 9.2, 2.6 Hz, 1H, Ar-H), 6.71 (s, 1H, Ar-H), 5.11 (t, *J* = 7.2 Hz, 1H, CH), 4.07 (dd, *J* = 17.6, 7.7 Hz, 1H, CH_2(a)_), 3.92 (dd, *J* = 17.6, 7.1 Hz, 1H, CH_2(b)_); ^13^C-NMR (101 MHz, DMSO-*d*_6_) δ 196.7 (CO), 160.7, 160.0 & 157.7 (C_1_-F, *J*_C-F_ = 233.3 Hz), 153.9, 138.3, 136.3 & 136.1 (C_3_-F, *J*_C-F_ = 12.6 Hz), 135.2, 130.0, 128.8, 128.4, 123.6 & 123.4 (C_4_-F, *J*_C-F_ = 2.4 Hz), 122.9, 122.6, 120.6, 119.8 & 119.7 (C_5_-F, *J*_C-F_ = 10.1 Hz), 114.5, 110.7, 107.2 & 106.9 (C_2_-F, *J*_C-F_ = 24.3 Hz), 101.9, 97.6 & 97.4 (C_6_-F, *J*_C-F_ = 25.2 Hz), 41.9 (CH), 31.7 (CH_2_); IR (KBr, cm^−1^) ν_max_ = 3304, 3028, 2910, 2850, 1665, 1554, 1450, 1394, 1363, 1338, 1252; [Anal. Calcd. for C_25_H_17_ClFNO_2_: C, 71.86; H, 4.10; N, 3.35; Found: C, 71.69; H, 4.25; N, 3.19]; LC/MS (ESI, *m*/*z*): 418.10 [M + H] ^+^, exact mass 417.09 for C_25_H_17_ClFNO_2_.

### 3.9. Synthesis of 3-(Benzo[b]thiophen-2-yl)-1-(4-bromophenyl)-3-(1H-indol-3-yl)propan-1-one (***3h***)

Following the general procedure (GP1) chalcone **1i** (172 mg, 0.5 mmol), indole **2a** (108 mg, 0.55 mmol) produces Friedel–Crafts product-**3h** (yield 156 mg, 68%); m.p. 121–122 °C; ^1^H-NMR (400 MHz, DMSO-*d*_6_) δ 10.97 (s, 1H, NH), 7.98 (d, *J* = 8.2 Hz, 2H, Ar-H), 7.72 (dt, *J* = 18.7, 9.0 Hz, 4H, Ar-H), 7.54 (d, *J* = 8.0 Hz, 1H, Ar-H), 7.41 (d, *J* = 2.5 Hz, 1H, Ar-H), 7.35–7.19 (m, 4H, Ar-H), 7.05 (t, *J* = 7.6 Hz, 1H, Ar-H), 6.92 (t, *J* = 7.4 Hz, 1H, Ar-H), 5.23 (t, *J* = 7.3 Hz, 1H, CH), 4.01 (dd, *J* = 10.8, 7.1 Hz, 2H, CH_2_); ^13^C-NMR (126 MHz, DMSO-*d*_6_) δ 197.0 (CO), 150.76, 139.5, 138.5, 136.4, 135.6, 131.8, 130.2, 127.5, 126.1, 124.1, 123.6, 123.0, 122.5, 122.2, 121.2, 120.2, 118.7, 118.5, 116.8, 111.5, 44.5 (CH), 33.4 (CH_2_); IR (KBr, cm^−1^) ν_max_ = 3335, 3038, 2907, 2852, 1678, 1580, 1455, 1440, 1360, 1348, 1255; [Anal. Calcd. for C_25_H_18_BrNOS: C, 65.22; H, 3.94; N, 3.04; Found: C, 65.04; H, 3.86; N, 2.91]; LC/MS (ESI, *m*/*z*): 460.04 [M + H] ^+^, exact mass 459.03 for C_25_H_18_BrNOS.

### 3.10. Synthesis of 3-(Benzofuran-2-yl)-3-(6-fluoro-1H-indol-3-yl)-1-(2-nitrophenyl)propan-1-one (***3i***)

Following the general procedure (GP1) chalcone **1e** (147 mg, 0.5 mmol) and 6-fluoroindole **2c** (74 mg, 0.55 mmol) produces Friedel–Crafts product-**3i** (yield 146 mg, 68%); m.p. 139–140 °C; ^1^H-NMR (500 MHz, DMSO-*d*_6_) δ 11.10 (d, *J* = 2.5 Hz, 1H, NH), 8.06 (dd, *J* = 8.0, 1.2 Hz, 1H, Ar-H), 7.78 (td, *J* = 7.5, 1.2 Hz, 1H, Ar-H), 7.71 (ddd, *J* = 12.9, 7.5, 1.6 Hz, 2H, Ar-H), 7.61 (dd, *J* = 8.8, 5.4 Hz, 1H, Ar-H), 7.54–7.50 (m, 1H, Ar-H), 7.44 (dt, *J* = 7.2, 1.3 Hz, 1H, Ar-H), 7.40 (d, *J* = 2.5 Hz, 1H, Ar-H), 7.22–7.12 (m, 3H, Ar-H), 6.83 (ddd, *J* = 9.7, 8.7, 2.4 Hz, 1H, Ar-H), 6.78–6.74 (m, 1H, Ar-H), 5.05 (t, *J* = 7.1 Hz, 1H, CH), 4.01 (dd, *J* = 17.9, 7.6 Hz, 1H, CH_2(a)_), 3.82 (dd, *J* = 18.0, 6.6 Hz, 1H, CH_2(b)_); ^13^C-NMR (126 MHz, DMSO-*d*_6_) δ 199.5 (CO), 160.1, 159.8 &157.9 (C_1_-F, *J*_C-F_ = 186.4 Hz), 154.0, 146.2, 136.3 & 136.2 (C_3_-F, *J*_C-F_ = 10.0 Hz), 135.4, 134.1, 131.8, 128.4 & 128.4 (C_4_-F, *J*_C-F_ = 3.8 Hz), 124.3, 123.9, 123.8, 123.5, 122.9, 122.7, 120.7, 119.7 & 119.6(C_5_-F, *J*_C-F_ = 8.0 Hz), 114.1, 110.8, 107.3 & 107.1 (C_2_-F, *J*_C-F_ = 19.5 Hz), 102.3, 97.7 & 97.5 (C_6_-F, *J*_C-F_ = 20.0 Hz), 45.2 (CH), 31.6 (CH_2_); IR (KBr, cm^−1^) ν_max_ = 3305, 3062,2915, 2858, 1699, 1621, 1527, 1452, 1338, 1255, 1144, 798, 748, 696; [Anal. Calcd. for C_25_H_17_FN_2_O_4_: C, 70.09; H, 4.00; N, 6.54; Found: C, 69.89; H, 4.11; N, 6.45]; LC/MS (ESI, *m*/*z*): 429.13 [M + H] ^+^, exact mass 428.12 for C_25_H_17_FN_2_O_4_.

### 3.11. Synthesis of 3-(Benzofuran-2-yl)-3-(5-bromo-1H-indol-3-yl)-1-(2-nitrophenyl)propan-1-one (***3j***)

Following the general procedure (**GP1**) chalcone **1e** (147 mg, 0.5 mmol) 5-bromoindole **2b** (108 mg, 0.55 mmol) produces Friedel–Crafts product-**3j** (yield 154 mg, 63%); m.p. 173–174 °C; ^1^H-NMR (400 MHz, DMSO-*d*_6_) δ 11.25 (d, *J* = 2.6 Hz, 1H, NH), 8.06 (d, *J* = 7.9 Hz, 1H, Ar-H), 7.84–7.76 (m, 2H, Ar-H), 7.73 (d, *J* = 7.6 Hz, 2H, Ar-H), 7.54 (d, *J* = 6.8 Hz, 1H, Ar-H), 7.50–7.40 (m, 2H, Ar-H), 7.33 (d, *J* = 8.7 Hz, 1H, Ar-H), 7.17 (q, *J* = 7.3, 6.7 Hz, 3H, Ar-H), 6.77 (s, 1H, Ar-H), 5.06 (t, *J* = 6.9 Hz, 1H, CH), 4.02 (dd, *J* = 18.0, 7.5 Hz, 1H, CH_2(a)_), 3.83 (dd, *J* = 17.8, 6.7 Hz, 1H, CH_2(b)_); ^13^C-NMR (126 MHz, DMSO-*d*_6_) δ 199.4 (CO), 159.9, 153.9, 146.1, 135.3, 134.9, 133.9, 131.7, 128.4, 128.4, 127.8, 124.9, 124.2, 123.7, 123.5, 122.6, 120.8, 120.6, 113.7, 113.6, 111.3, 110.7, 102.2, 45.1 (CH), 31.2 (CH_2_); IR (KBr, cm^−1^) ν_max_ = 3324, 3062, 2918, 2850, 1695, 1570, 1519, 1452, 1334, 1245, 1099, 879, 854, 791, 746, 694, 604; [Anal. Calcd. for C_25_H_17_BrN_2_O_4_: C, 61.36; H, 3.50; N, 5.72; Found: C, 61.21; H, 3.63; N, 5.62]; LC/MS (ESI, *m*/*z*): 489.05 [M + H], exact mass 488.04 for C_25_H_17_BrN_2_O_4_.

### 3.12. Synthesis of 3-(Benzofuran-2-yl)-3-(1H-indol-3-yl)-1-(2-nitrophenyl)propan-1-one (***3k***)

Following the general procedure (GP1) chalcone **1e** (147 mg, 0.5 mmol) indole **2a** (64 mg, 0.55 mmol) produces Friedel–Crafts product-**3k** (yield 135 mg, 66%); m.p. 154–155 °C; ^1^H-NMR (500 MHz, DMSO-*d*_6_) δ 11.01 (d, *J* = 2.5 Hz, 1H, NH), 8.06 (dd, *J* = 8.0, 1.2 Hz, 1H, Ar-H), 7.78 (td, *J* = 7.5, 1.2 Hz, 1H, Ar-H), 7.74–7.68 (m, 2H, Ar-H), 7.60 (d, *J* = 7.9 Hz, 1H, Ar-H), 7.54–7.50 (m, 1H, Ar-H), 7.45–7.41 (m, 1H, Ar-H), 7.39–7.33 (m, 2H, Ar-H), 7.17 (pd, *J* = 7.2, 1.4 Hz, 2H, Ar-H), 7.08–7.03 (m, 1H, Ar-H), 6.95 (t, *J* = 7.4 Hz, 1H, Ar-H), 6.75 (s, 1H, Ar-H), 5.05 (t, *J* = 7.1 Hz, 1H, CH), 4.01 (dd, *J* = 17.9, 7.8 Hz, 1H, CH_2(a)_), 3.82 (dd, *J* = 17.9, 6.4 Hz, 1H, CH_2(b)_); ^13^C-NMR (126 MHz, DMSO-*d*_6_) δ 199.6 (CO), 160.3, 153.9, 146.2, 136.4, 135.4, 134.1, 131.8, 128.5, 128.4, 126.0, 124.3, 123.5, 123.2, 122.7, 121.2, 120.7, 118.7, 118.6, 113.8, 111.7, 110.8, 102.2, 45.3 (CH), 31.6 (CH_2_); IR (KBr, cm^−1^) ν_max_ = 3310, 3055, 2901, 2850, 1684, 1625, 1530, 145, 1331, 1253; [Anal. Calcd. for C_25_H_18_N_2_O_4_: C, 73.16; H, 4.42; N, 6.83; Found: C, 73.10; H, 4.38; N, 6.79]; LC/MS (ESI, *m*/*z*): 411.13 [M + H] ^+^, exact mass 410.13 for C_25_H_18_N_2_O_4_.

### 3.13. Synthesis of 3-(Benzofuran-2-yl)-3-(1H-indol-3-yl)-1-(3-methoxyphenyl)propan-1-one (***3l***)

Following the general procedure (GP1) chalcone **1g** (139 mg, 0.5 mmol) indole **2a** (64 mg, 0.55 mmol) produces Friedel–Crafts product-**3l** (yield 162 mg, 82%); m.p. 162–163 °C; ^1^H-NMR (500 MHz, DMSO-*d*_6_) δ 10.97 (d, *J* = 2.5 Hz, 1H, NH), 7.68–7.61 (m, 2H, Ar-H), 7.52–7.47 (m, 2H, Ar-H), 7.46–7.40 (m, 2H, Ar-H), 7.37–7.33 (m, 2H, Ar-H), 7.21–7.14 (m, 3H, Ar-H), 7.05 (ddd, *J* = 8.2, 7.0, 1.2 Hz, 1H, Ar-H), 6.95 (ddd, *J* = 8.0, 7.0, 1.0 Hz, 1H, Ar-H), 6.70 (d, *J* = 1.0 Hz, 1H, Ar-H), 5.10 (t, *J* = 7.2 Hz, 1H, CH), 4.04 (dd, *J* = 17.5, 7.5 Hz, 1H, CH_2(a)_), 3.93 (dd, *J* = 17.5, 7.0 Hz, 1H, CH_2(b)_), 3.79 (s, 3H, OCH_3_); ^13^C-NMR (126 MHz, DMSO-*d*_6_) δ 197.5 (CO), 161.0, 159.4, 153.9, 138.0, 136.3, 129.9, 128.4, 126.1, 123.3, 123.0, 122.5, 121.1, 120.6, 120.5, 119.4, 118.7, 118.5, 114.2, 112.4, 111.6, 110.7, 101.8, 55.3 (OCH_3_), 42.0 (CH), 31.8 (CH_2_); IR (KBr, cm^−1^) ν_max_ = 3392, 3055, 2960, 2916, 2852, 1674, 1591, 1452,1423, 1333, 1265, 1190, 1161, 1095, 1008, 813, 790, 744, 609, 544; [Anal. Calcd. for C_26_H_21_NO_3_: C, 78.97; H, 5.35; N, 3.54; Found: C, 79.13; H, 5.25; N, 3.44]; LC/MS (ESI, *m*/*z*): 396.16 [M + H], exact mass 395.15 for C_26_H_21_NO_3_.

### 3.14. Synthesis of 3-(Benzofuran-2-yl)-3-(6-fluoro-1H-indol-3-yl)-1-(3-methoxyphenyl)propan-1-one (***3m***)

Following the general procedure (GP1) chalcone **1g** (139 mg, 0.5 mmol) 6- fluoroindole **2c** (74 mg, 0.55 mmol) produces Friedel–Crafts product-**3m** (yield 153 mg, 74%); m.p. 154–155 °C; ^1^H-NMR (400 MHz, DMSO-*d*_6_) δ 11.04 (d, *J* = 2.4 Hz, 1H, NH), 7.67–7.58 (m, 2H, Ar-H), 7.53–7.46 (m, 2H, Ar-H), 7.46–7.40 (m, 2H, Ar-H), 7.36 (d, *J* = 2.5 Hz, 1H, Ar-H), 7.22–7.09 (m, 4H, Ar-H), 6.82 (td, *J* = 9.4, 2.5 Hz, 1H, Ar-H), 6.71 (s, 1H, Ar-H), 5.08 (t, *J* = 7.1 Hz, 1H, CH), 4.03 (dd, *J* = 17.5, 7.5 Hz, 1H, CH_2__(a)_), 3.95–3.87 (m, 1H, CH_2__(b)_), 3.79 (s, 3H, CH_3_); ^13^C-NMR (126 MHz, DMSO-*d*_6_) δ 197.5 (CO), 160.8, 159.4, 159.78 &157.9(C_1_-F, *J*_C-F_ = 186.0 Hz), 153.9, 137.9, 136.2 & 136.1 (C_3_-F, *J*_C-F_ = 10.1 Hz), 129.9, 128.4, 123.7, 123.4, 123.0, 122.6, 120.6 & 120.6 (C_4_-F, *J*_C-F_ = 7.4 Hz), 119.7 & 119.6 (C_5_-F, *J*_C-F_ = 8.1 Hz), 119.5, 114.5, 112.5, 110.7, 107.2 & 106.9 (C_2_-F, *J*_C-F_ = 19.4 Hz), 101.9, 97.6 & 97.4 (C_6_-F, *J*_C-F_ = 20.3 Hz), 55.3 (OCH_3_), 42.0 (CH), 31.7 (CH_2_); IR (KBr, cm^−1^) ν_max_ = 3409, 3053, 2922, 2836, 1666, 1513, 1483, 1454, 1430, 1286, 1244, 1163, 1101, 1016, 806, 740, 586; [Anal. Calcd. for C_26_H_20_FNO_3_: C, 75.53; H, 4.88; N, 3.39; Found: C, 75.41; H, 4.99; N, 3.27]; LC/MS (ESI, *m*/*z*): 414.15 [M + H] ^+^, exact mass 413.14 for C_26_H_20_FNO_3_.

### 3.15. Synthesis of 3-(Benzo[b]thiophen-2-yl)-3-(1H-indol-3-yl)-1-phenylpropan-1-one (***3n***)

Following the general procedure (GP1) chalcone **1h** (132 mg, 0.5 mmol) indole **2a** (64 mg, 0.55 mmol) produces Friedel–Crafts product-**3n** (yield 164 mg, 86%); m.p. 159–160 °C; ^1^H-NMR (400 MHz, DMSO-*d*_6_) δ 10.90 (s, 1H, NH), 7.97 (d, *J* = 7.7 Hz, 2H, Ar-H), 7.68 (d, *J* = 8.1 Hz, 1H, Ar-H), 7.60 (d, *J* = 8.0 Hz, 1H, Ar-H), 7.54 (t, *J* = 7.4 Hz, 1H, Ar-H), 7.50–7.40 (m, 3H, Ar-H), 7.35 (s, 1H, Ar-H), 7.30–7.22 (m, 2H, Ar-H), 7.19 (t, *J* = 7.5 Hz, 1H, Ar-H), 7.13 (t, *J* = 7.6 Hz, 1H, Ar-H), 6.98 (t, *J* = 7.7 Hz, 1H, Ar-H), 6.86 (t, *J* = 7.5 Hz, 1H, Ar-H), 5.19 (t, *J* = 7.2 Hz, 1H, CH), 4.02–3.87 (m, 2H, CH_2_); ^13^C-NMR (101 MHz, DMSO-*d*_6_) δ 197.8 (CO), 150.9, 139.6, 138.6, 136.7, 136.475, 133.4, 128.7, 128.1, 126.2, 124.1, 123.7, 122.9, 122.4, 122.1, 121.1, 120.2, 118.8, 118.6, 116.9, 111.5, 44.6 (CH), 33.4 (CH_2_); IR (KBr, cm^−1^) ν_max_ = 3427, 3394, 3053, 2885, 1668, 1597, 1450, 1334, 1265, 1194; [Anal. Calcd. for C_25_H_19_NOS: C, 78.71; H, 5.02; N, 3.67; Found: C, 78.62; H, 5.17; N, 3.61]; LC/MS (ESI, *m*/*z*): 382.13 [M + H] ^+^, exact mass 381.12 for C_25_H_19_NOS.

### 3.16. Synthesis of 3-(Benzo[b]thiophen-2-yl)-1-(4-chlorophenyl)-3-(1H-indol-3-yl)propan-1-one (***3o***)

Following the general procedure (GP1) chalcone **1j** (149 mg, 0.5 mmol) indole **2a** (64 mg, 0.55 mmol) produces Friedel–Crafts product-**3o** (yield 160 mg, 77%); m.p. 150–151 °C; ^1^H-NMR (400 MHz, DMSO-*d*_6_) δ 10.99 (d, *J* = 2.5 Hz, 1H, NH), 8.09–8.03 (m, 2H, Ar-H), 7.76 (d, *J* = 8.0 Hz, 1H, Ar-H), 7.69 (d, *J* = 7.9 Hz, 1H, Ar-H), 7.57 (dd, *J* = 11.7, 8.4 Hz, 3H, Ar-H), 7.43 (d, *J* = 2.7 Hz, 1H, Ar-H), 7.35 (d, *J* = 8.2 Hz, 1H, Ar-H), 7.32 (s, 1H, Ar-H), 7.26 (d, *J* = 7.9 Hz, 1H, Ar-H), 7.22 (d, *J* = 7.4 Hz, 1H, Ar-H), 7.06 (t, *J* = 7.6 Hz, 1H, Ar-H), 6.94 (t, *J* = 7.5 Hz, 1H, Ar-H), 5.25 (t, *J* = 7.1 Hz, 1H, CH), 4.11–3.95 (m, 2H, CH_2_); ^13^C-NMR (101 MHz, DMSO-*d*_6_) δ 196.8 (CO), 150.8, 139.6, 138.5, 138.2, 136.4, 135.3, 130.1, 128.8, 126.1, 124.1, 123.5, 123.1, 122.6, 122.2, 121.1, 120.3, 118.7, 118.6, 116.8, 111.6, 44.6 (CH), 33.4 (CH_2_).; IR (KBr, cm^−1^) ν_max_ = 3383, 3055, 2954, 2921, 2852, 1676, 1583, 1483, 1454, 1402, 1356, 1328, 1261, 1194; [Anal. Calcd. for C_25_H_18_ClNOS: C, 72.19; H, 4.36; N, 3.37; Found: C, 72.05; H, 4.24; N, 3.33]; LC/MS (ESI, *m*/*z*): 416.10 [M + H] ^+^, exact mass 415.08 for C_25_H_18_ClNOS.

### 3.17. Synthesis of 3-(Benzo[b]thiophen-2-yl)-1-(4-fluorophenyl)-3-(1H-indol-3-yl)propan-1-one (***3p***)

Following the general procedure (GP1) chalcone **1k** (141 mg, 0.5 mmol) indole **2a** (64 mg, 0.55 mmol) produces Friedel–Crafts product-**3p** (yield 164 mg, 82%); m.p. 142–143 °C; ^1^H-NMR (400 MHz, DMSO-*d*_6_) δ 10.98 (s, 1H, NH), 8.14 (dd, *J* = 8.5, 5.6 Hz, 2H, Ar-H), 7.76 (d, *J* = 8.0 Hz, 1H, Ar-H), 7.68 (d, *J* = 7.9 Hz, 1H, Ar-H), 7.55 (d, *J* = 8.0 Hz, 1H, Ar-H), 7.43 (s, 1H, Ar-H), 7.40–7.30 (m, 4H, Ar-H), 7.30–7.24 (m, 1H, Ar-H), 7.21 (t, *J* = 7.6 Hz, 1H, Ar-H), 7.06 (t, *J* = 7.6 Hz, 1H, Ar-H), 6.93 (t, *J* = 7.5 Hz, 1H, Ar-H), 5.25 (t, *J* = 7.2 Hz, 1H, CH), 4.02 (dd, *J* = 11.1, 7.2 Hz, 2H, CH_2_); ^13^C-NMR (101 MHz, DMSO-*d*_6_) δ 196.4 (CO), 166.3 & 163.8 (C_1_-F, *J*_C-F_ = 250.2 Hz), 150.8, 139.6, 138.5, 136.4, 133.4 & 133.4 (C_4_-F, *J*_C-F_ = 2.9 Hz), 131.2, 126.1, 124.1, 123.6, 123.0, 122.5, 122.5, 122.2, 121.1, 120.2 & 120.2 (C_3_-F, *J*_C-F_ = 6.3 Hz), 118.5, 116.9, 115.8 & 115.6 (C_2_-F, *J*_C-F_ = 22.0 Hz), 111.5, 44.5 (CH), 33.5(CH_2_); IR (KBr, cm^−1^) ν_max_ = 3400, 3059, 2904, 1670, 1591, 1502, 1454, 1411, 1356, 1335, 1226, 1153; [Anal. Calcd. for C_25_H_18_FNOS: C, 75.16; H, 4.54; N, 3.51; Found: C, 75.26; H, 4.43; N, 3.47]; LC/MS (ESI, *m*/*z*): 400.13 [M + H] ^+^, exact mass 399.11 for C_25_H_18_FNOS.

### 3.18. Synthesis of 3-(Benzo[b]thiophen-2-yl)-3-(1H-indol-3-yl)-1-(4-(trifluoromethyl)phenyl)propan-1-one (***3q***)

Following the general procedure (GP1) chalcone **1l** (162 mg, 0.5 mmol) indole **2a** (64 mg, 0.55 mmol) produces Friedel–Crafts product-**3q** (yield 171 mg, 76%); m.p. 165–166 °C; ^1^H-NMR (400 MHz, DMSO-*d*_6_) δ 10.98 (d, *J* = 2.5 Hz, 1H, NH), 8.23 (d, *J* = 8.1 Hz, 2H, Ar-H), 7.89 (d, *J* = 8.3 Hz, 2H, Ar-H), 7.77 (d, *J* = 8.0 Hz, 1H, Ar-H), 7.69 (d, *J* = 7.9 Hz, 1H, Ar-H), 7.56 (d, *J* = 8.0 Hz, 1H, Ar-H), 7.43 (d, *J* = 2.6 Hz, 1H, Ar-H), 7.35 (d, *J* = 8.6 Hz, 2H, Ar-H), 7.30–7.19 (m, 2H, Ar-H), 7.06 (t, *J* = 7.6 Hz, 1H, Ar-H), 6.94 (t, *J* = 7.4 Hz, 1H, Ar-H), 5.26 (t, *J* = 7.1 Hz, 1H, CH), 4.10 (t, *J* = 7.4 Hz, 2H, CH_2_); ^13^C-NMR (101 MHz, DMSO-*d*_6_) δ 197.3 (CO), 150.6, 139.7, 139.5, 138.5, 136.4, 132.7, 132.4, 128.9, 126.1, 125.70, 124.1, 123.6, 123.0, 122.5, 122.1, 121.2, 120.2, 118.6, 116.7, 111.5, 44.9 (CH), 33.4 (CH_2_); IR (KBr, cm^−1^) ν_max_ = 3458, 3415, 3050, 2960, 2902, 1689, 1454, 1409, 1319, 1261, 1166; [Anal. Calcd. for C_26_H_18_F_3_NOS: C, 69.47; H, 4.04; N, 3.12; Found: C, 69.59; H, 3.97; N, 3.07]; LC/MS (ESI, *m*/*z*): 450.11 [M + H] ^+^, exact mass 449.11 for C_26_H_18_F_3_NOS.

### 3.19. Synthesis of 3-(Benzofuran-2-yl)-3-(1H-indol-3-yl)-1-(2-methoxyphenyl)propan-1-one (***3r***):

Following the general procedure (GP1) chalcone **1f** (139 mg, 0.5 mmol) indole **2a** (64 mg, 0.55 mmol) produces Friedel–Crafts product-**3r** (yield 158 mg, 80%); m.p. 67–68 °C; ^1^H-NMR (400 MHz, DMSO-*d*_6_) δ 10.96 (d, *J* = 2.5 Hz, 1H, NH), 7.56–7.48 (m, 3H, Ar-H), 7.47–7.40 (m, 2H, Ar-H), 7.35 (d, *J* = 8.1 Hz, 1H, Ar-H), 7.26 (d, *J* = 2.6 Hz, 1H, Ar-H), 7.16 (dd, *J* = 8.1, 4.9 Hz, 3H, Ar-H), 7.06 (t, *J* = 7.4 Hz, 1H, Ar-H), 6.96 (dt, *J* = 14.9, 7.2 Hz, 2H, Ar-H), 6.62 (s, 1H, Ar-H), 5.06 (t, *J* = 7.2 Hz, 1H, CH), 3.94 (dd, *J* = 17.1, 7.6 Hz, 1H, CH_2(a)_), 3.86 (s, 3H, OCH_3_), 3.78 (dd, *J* = 17.0, 7.1 Hz, 1H, CH_2(b)_); ^13^C-NMR (101 MHz, DMSO-*d*_6_) δ 199.5 (CO), 160.9, 158.0, 153.9, 136.3, 133.6, 129.6, 129.4, 128.4, 127.7, 126.0, 122.9, 122.7, 121.1, 120.6, 120.4, 118.6, 118.4, 114.2, 112.5, 111.7, 110.6, 101.9, 55.8 (OCH_3_), 47.1 (CH), 31.9 (CH_2_); IR (KBr, cm^−1^) ν_max_ = 3408, 3051, 2918, 2837, 1666, 1599, 1483, 1454, 1430, 1340, 1286, 1244, 1161; [Anal. Calcd. for C_26_H_21_NO_3_: C, 78.97; H, 5.35; N, 3.54; Found: C, 78.81; H, 5.19; N, 3.39]; LC/MS (ESI, *m*/*z*): 396.16 [M + H] ^+^, exact mass 395.15 for C_26_H_21_NO_3._

### 3.20. Synthesis of 3-(Benzofuran-2-yl)-3-(5-bromo-1H-indol-3-yl)-1-(2-methoxyphenyl)propan-1-one (***3s***)

Following the general procedure (GP1) chalcone **1f** (139 mg, 0.5 mmol) 5-bromoindole **2b** (108 mg, 0.55 mmol) produces Friedel–Crafts product-**3s** (yield 180 mg, 76%); m.p. 60–61 °C; ^1^H-NMR (400 MHz, DMSO-*d*_6_) δ 11.20 (d, *J* = 2.3 Hz, 1H, NH), 7.72 (s, 1H, Ar-H), 7.51 (t, *J* = 7.5 Hz, 2H, Ar-H), 7.47–7.41 (m, 2H, Ar-H), 7.37–7.31 (m, 2H, Ar-H), 7.17 (dd, *J* = 11.8, 7.6 Hz, 4H, Ar-H), 6.98 (t, *J* = 7.4 Hz, 1H, Ar-H), 6.65 (s, 1H, Ar-H), 5.05 (t, *J* = 7.3 Hz, 1H, CH), 3.91 (dd, *J* = 17.3, 7.4 Hz, 1H, CH_2(a)_), 3.87 (s, 3H, OCH_3_), 3.81 (dd, *J* = 17.1, 7.3 Hz, 1H, CH_2(b)_); ^13^C-NMR (126 MHz, DMSO-*d*_6_) δ 199.4, 160.6, 158.1, 153.9, 135.0, 133.7, 129.6, 128.3, 127.9, 127.6, 124.7, 123.6, 123.5, 122.6, 120.8, 120.6, 120.5, 114.0, 113.6, 112.4, 111.2, 110.7, 101.9, 55.8, 47.0, 31.8; IR (KBr, cm^−1^) ν_max_ = 3320, 3045, 2915, 2857, 1668, 1575, 1452, 1395, 1365, 1338, 1252; [Anal. Calcd. for C_26_H_20_BrNO_3_: C, 65.83; H, 4.25; N, 2.95; Found: C, 66.02; H, 4.11; N, 2.86]; LC/MS (ESI, *m*/*z*): 474.07 [M + H] ^+^, exact mass 473.06 for C_26_H_20_BrNO_3_

### 3.21. The Biological Activity Assays Protocols

Cytotoxicity against BJ Human fibroblast cells, and anti-cancer activity against PC3, HeLa and MCF-7 cancer cell lines were evaluated by following the procedure as described in the literature [25,26,27,28,29] (Supplementary Materials).

## 4. Conclusions

We have successfully synthesized multi-scaffold-based indoles, benzofurans and benzothiophenes. The anti-cancer activity showed promising results which make them candidates for further research. Fluorine atoms apparently play a crucial role in the bioactivity of this class of compounds with **3b** appearing to be the most active member of the series against cervical cancer HeLa (IC_50_ = 8.2 ± 0.2 µM) and breast cancer MCF-7 cell lines (IC_50_ = 12.3 ± 0.04 µM), whereas hit **3e** (IC_50_ = 7.8 ± 0.4 µM) appeared more active against PC3 prostate cancer cell line. The mechanism of action and in vivo study are required to further validate results of in vitro status.

## Figures and Tables

**Table 1:** The IC_50_ results of the synthesized against 4 cancer cell lines.

Compound	Structure 3a–3s	Cancer Type/Cell Line (IC_50_, µM)			
		Human fibroblast BJ	PC3	HeLa	MCF-7
**3a**	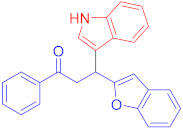	NA	11.4 ± 0.2	22.5 ± 0.9	NA
**3b**	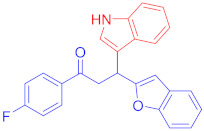	>30	22.2 ± 0.5	8.2 ± 0.2	12.37 ± 0.04
**3c**	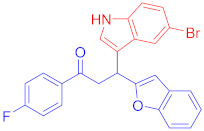	>30	21.9 ± 1.5	10.8 ± 0.1	25.87 ± 0.80
**3d**	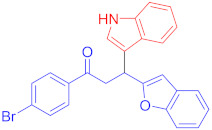	>30	26.3 ± 0.8	12.6 ± 0.4	NA
**3e**	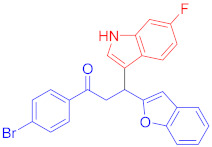	>30	7.8 ± 0.4	13.6 ± 0.4	NA
**3f**	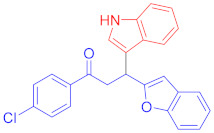	>30	27.0 ± 0.4	11.9 ± 0.2	19.28 ± 0.78
**3g**	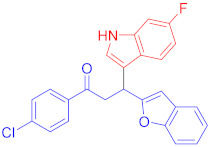	>30	20.0 ± 1.3	14.7 ± 1.1	NA
**3h**	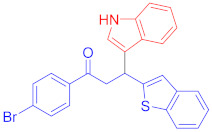	>30	29.7 ± 0.4	13.8 ± 0.3	NA
**3i**	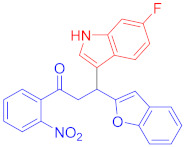	NA	NA	15.2 ± 0.6	NA
**3j**	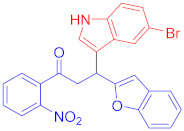	NA	16.0 ± 0.1	17.3 ± 0.7	NA
**3k**	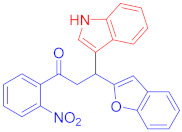	NA	NA	14.9 ± 0.6	NA
**3l**	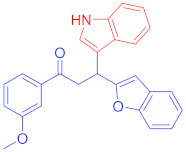	>30	16.3 ± 1.6	11.1 ± 0.6	NA
**3m**	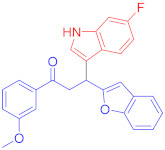	>50	19.3 ± 0.1	15.2 ± 0.2	22.93 ± 0.43
**3n**	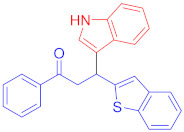	>50	20.4 ± 0.4	12.4 ± 0.2	NA
**3o**	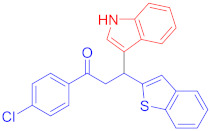	>50	29.7 ± 0.1	13.8 ± 0.3	NA
**3p**	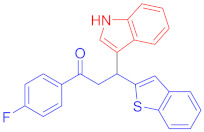	>50	27.6 ± 0.1	12.7 ± 0.1	NA
**3q**	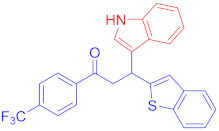	>50	18.0 ± 0.3	18.8 ± 0.2	NA
**3r**	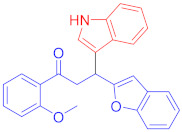	NA	NA	NA	NA
**3s**	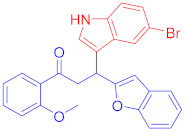	NA	NA	18.4 ± 0.2	28.64 ± 0.33
	STD. Doxorubicin	NA	1.9 ± 0.4	0.9 ± 0.14	0.79 ± 0.05

IC_50_ (*μ*M) was evaluated using MTT assay and ± is the standard deviation from three independent experiments. NA: means that the tested compound did not show anticancer activity at 30 µM.

## Data Availability

The data presented in this study are available in Supplementary Materials.

## Supplementary Materials

The following are available online, ^1^H-NMR and ^13^C-NMR for compounds **3a**–**3s** along with the biological activity assays protocols are provided in the Supplementary Materials.

## Sample Availability

Samples of the compounds **3a**–**3s** are available from the authors.
