# Supplementary material for: Bimetallic Iron–Palladium Catalyst System as a Lewis-Acid for the Synthesis of Novel Pharmacophores Based Indole Scaffold as Anticancer Agents
Source: Molecules. 2021 Apr 12;26(8):2212. doi: 10.3390/molecules26082212 (PMC8070033; doi:10.3390/molecules26082212)
Supplement: Supplementary file 1 [file molecules-26-02212-s001.pdf]

Bimetallic Iron–Palladium Catalyst System as a Lewis-Acid for the Synthesis of Novel Pharmacophores Based Indole Scaffold as Anticancer Agents

Mohammad Shahidul Islam [1], M. Ali [1], Abdullah Mohammed Al-Majid [1], Abdullah Saleh Alamary [1], Saeed Alshahrani [1], Sammer Yousuf [2], M. Iqbal Choudhary [2] and Assem Barakat [1,3,*]

[1] Department of Chemistry, College of Science, King Saud University, P. O. Box 2455, Riyadh 11451, Saudi Arabia; Emails: mislam@ksu.edu.sa (M.S.I.); maly.c@ksu.edu.sa (M.A); amajid@ksu.edu.sa (A.M.A.-M.); alamary1401@yahoo.com (A.S.A.); chemistry99y@gmail.com (S.A.).

[2] H.E.J. Research Institute of Chemistry, International Center for Chemical and Biological Sciences, University of Karachi, Karachi 75270, Pakistan; dr.sammer.yousuf@gmail.com (S.Y.); iqbal.choudhary@iccs.edu (M.I.C.)

[3] Department of Chemistry, Faculty of Science, Alexandria University, P.O. Box 426, Ibrahimia, Alexandria 21321, Egypt

* Correspondence: ambarakat@ksu.edu.sa; Tel.: +966-11467-5901; Fax: +966-11467-5992

NMR SPECTRA

^{1}H-NMR and ^{13}C-NMR for compound-3a

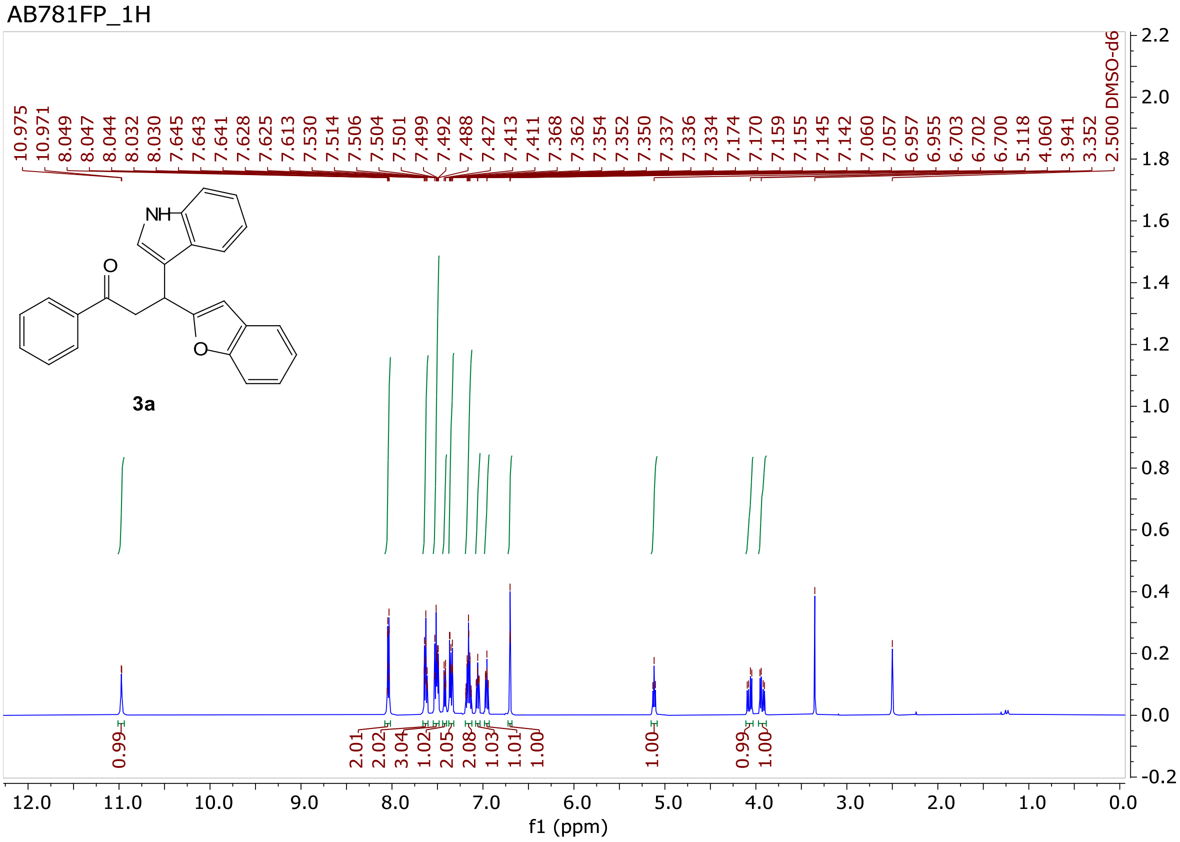

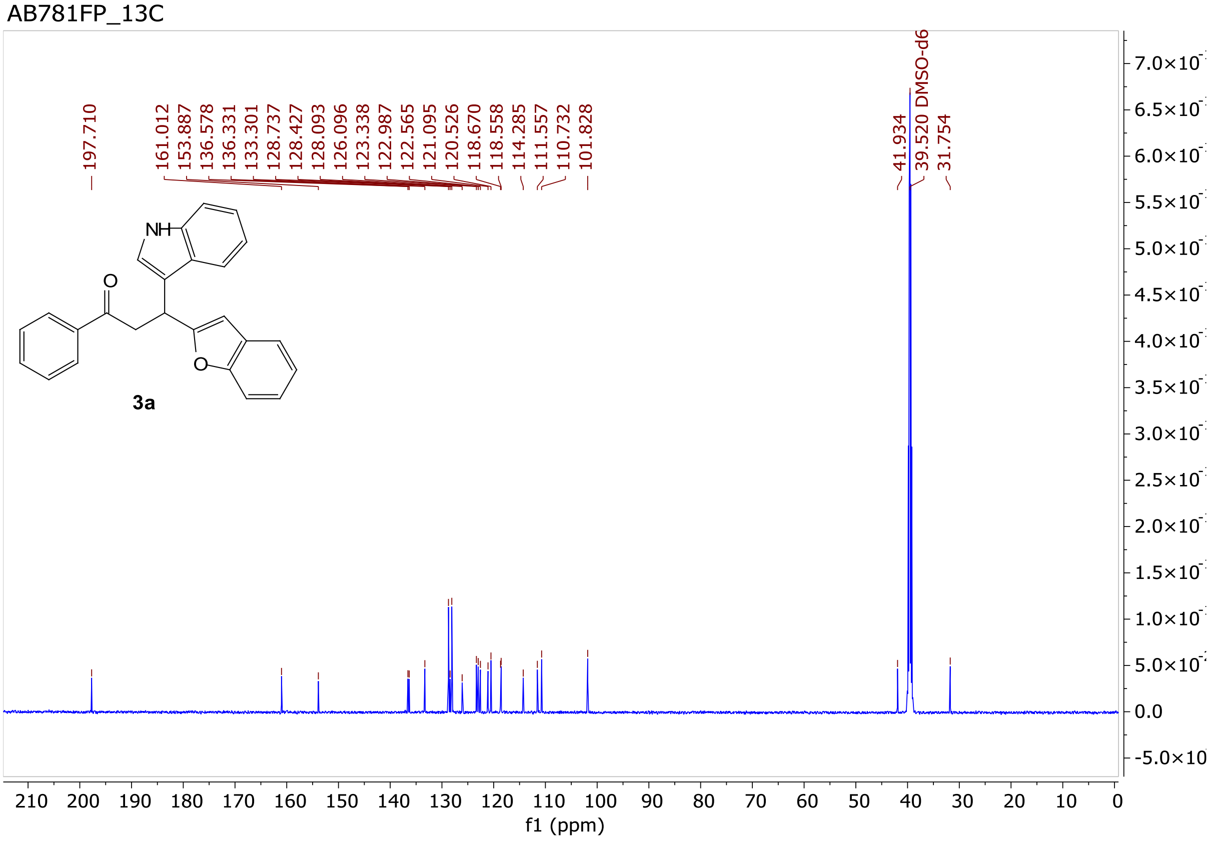

^{1}H-NMR and ^{13}C-NMR for compound-3b

AB793FP_1H

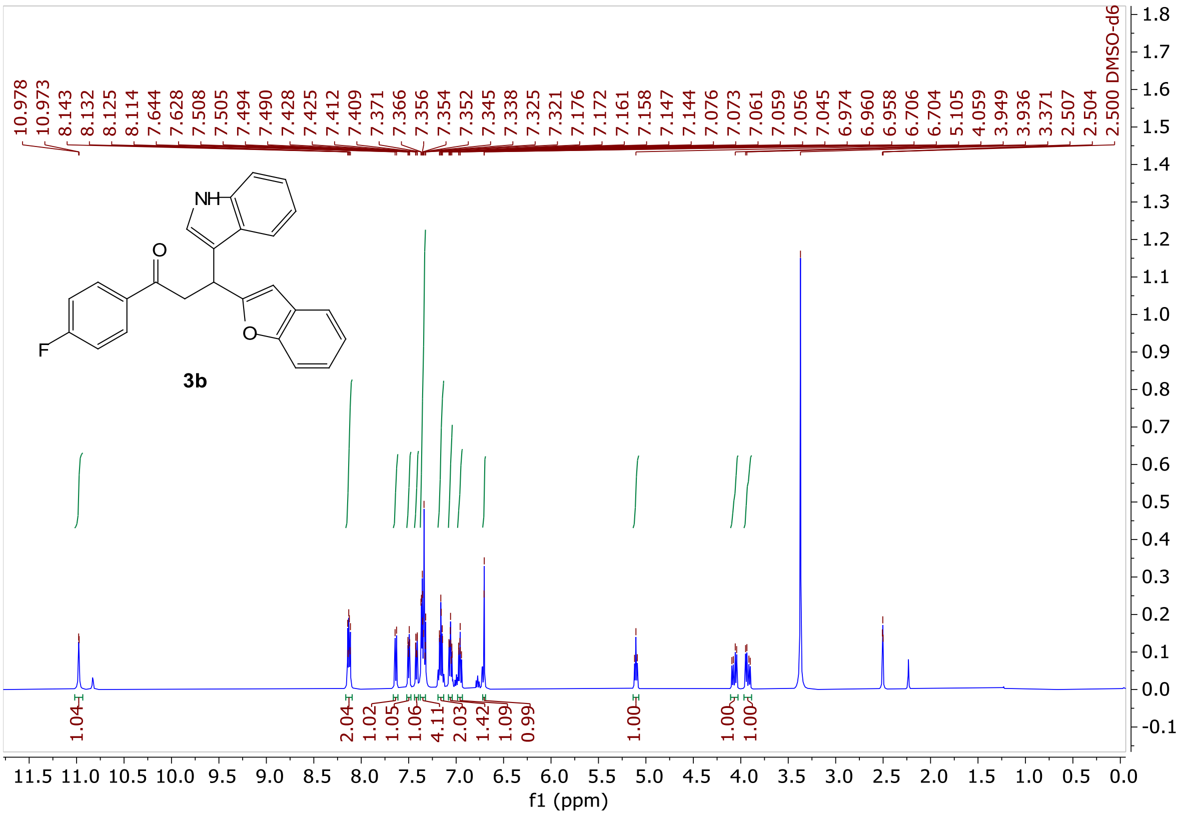

AB793FP_13C

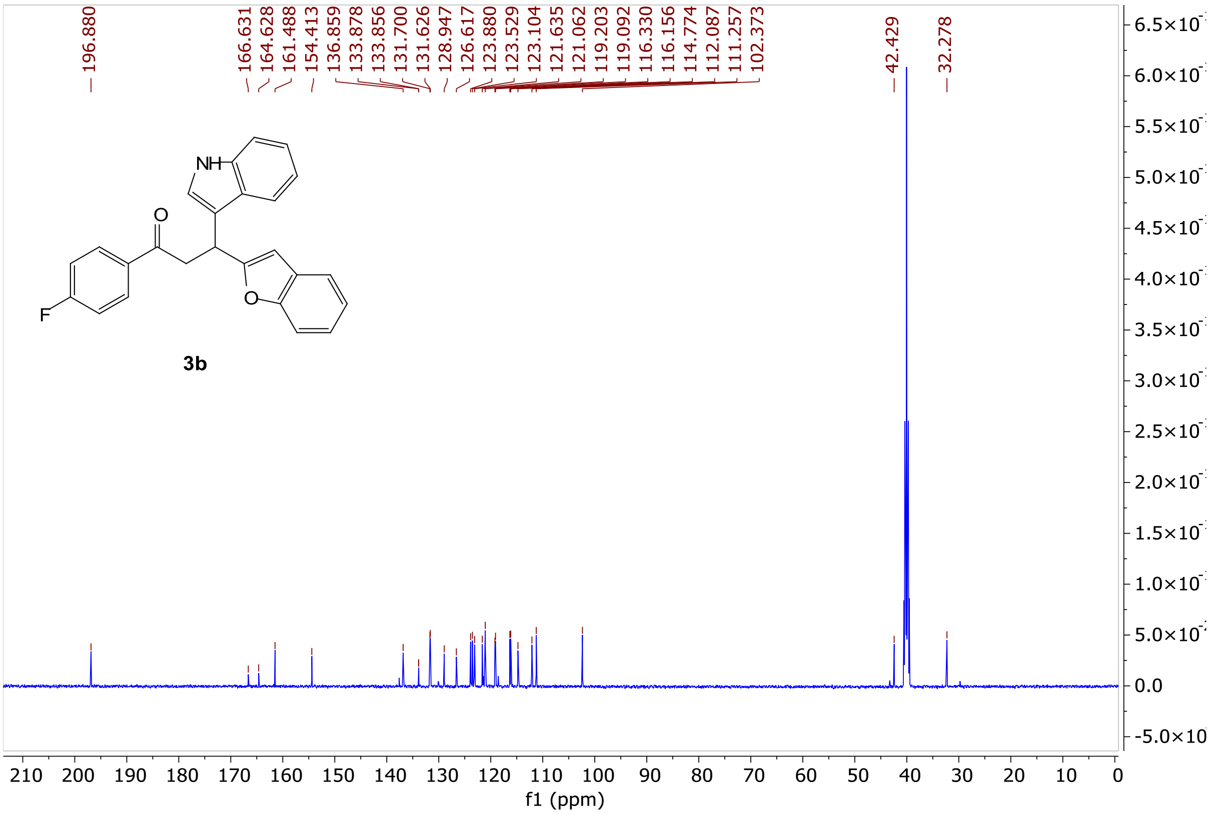

^{1}H-NMR and ^{13}C-NMR for compound-3c

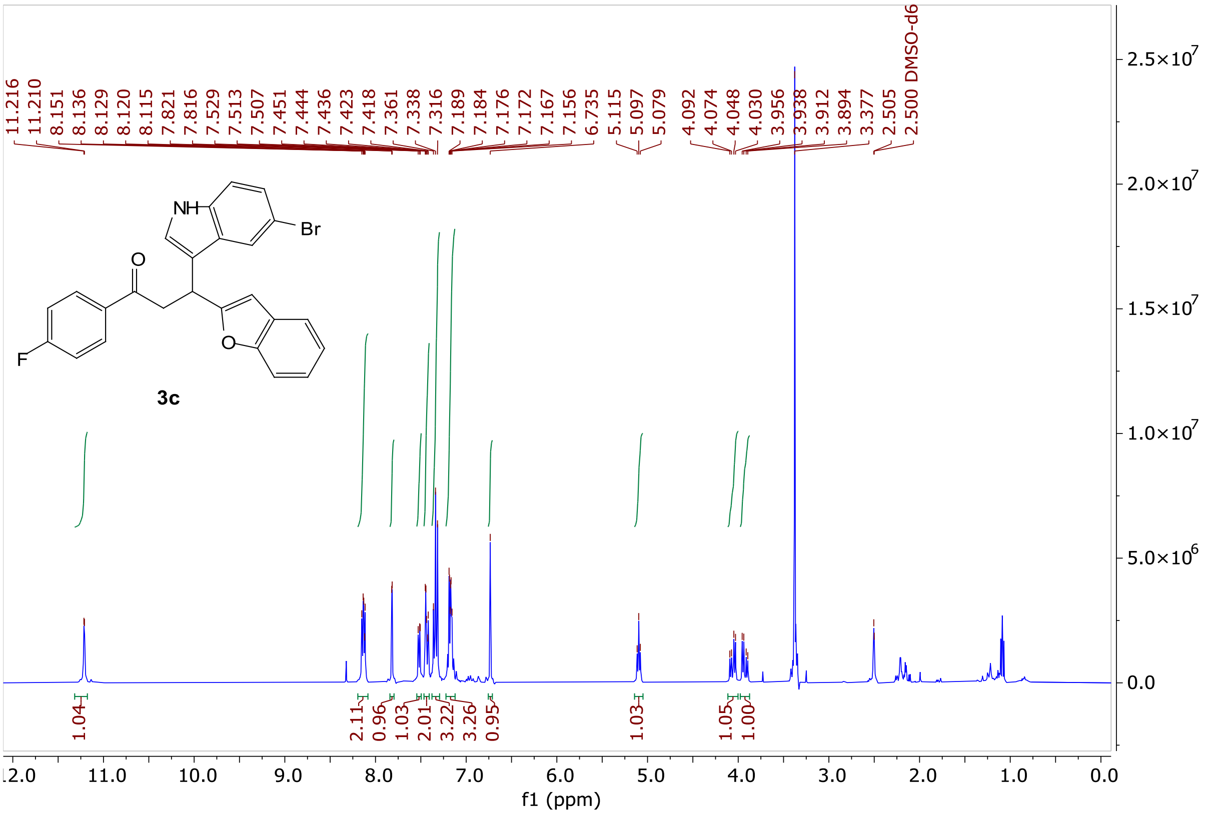

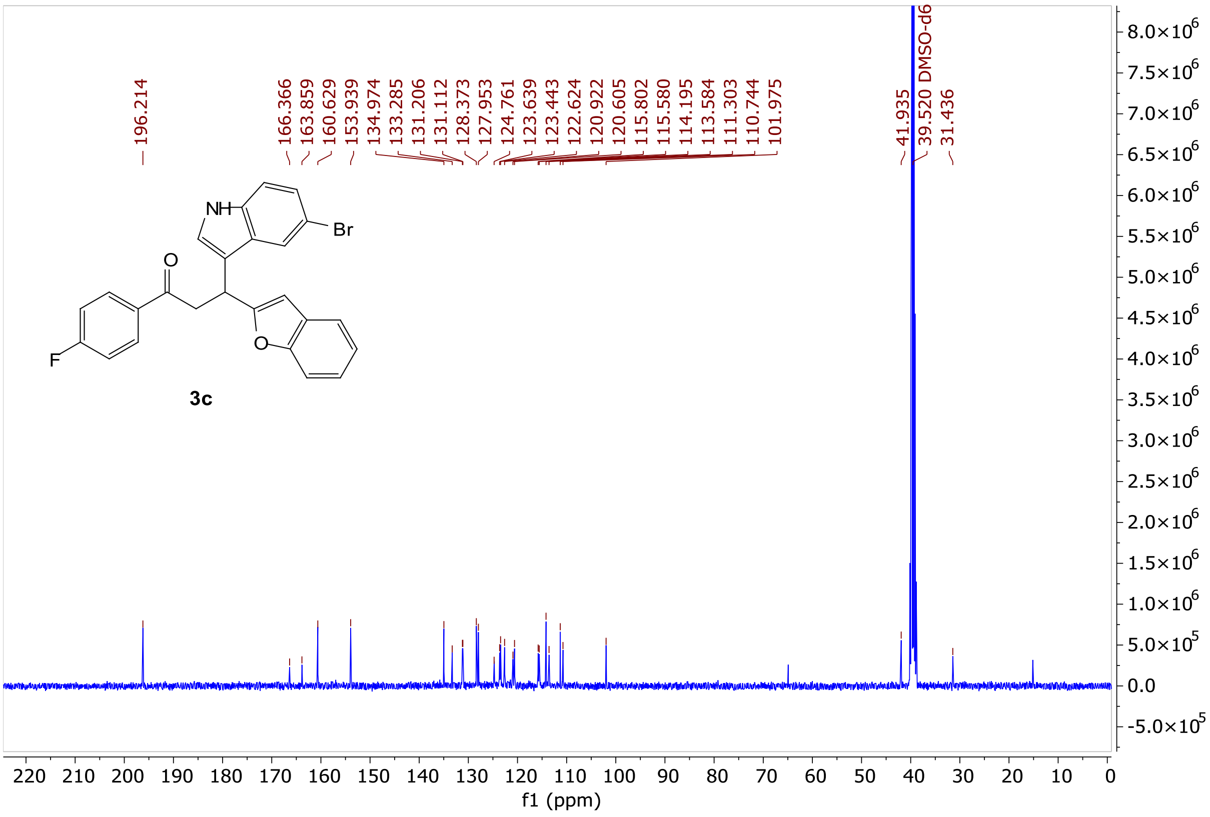

^{1}H-NMR and ^{13}C-NMR for compound-3d

AB799FP_1H

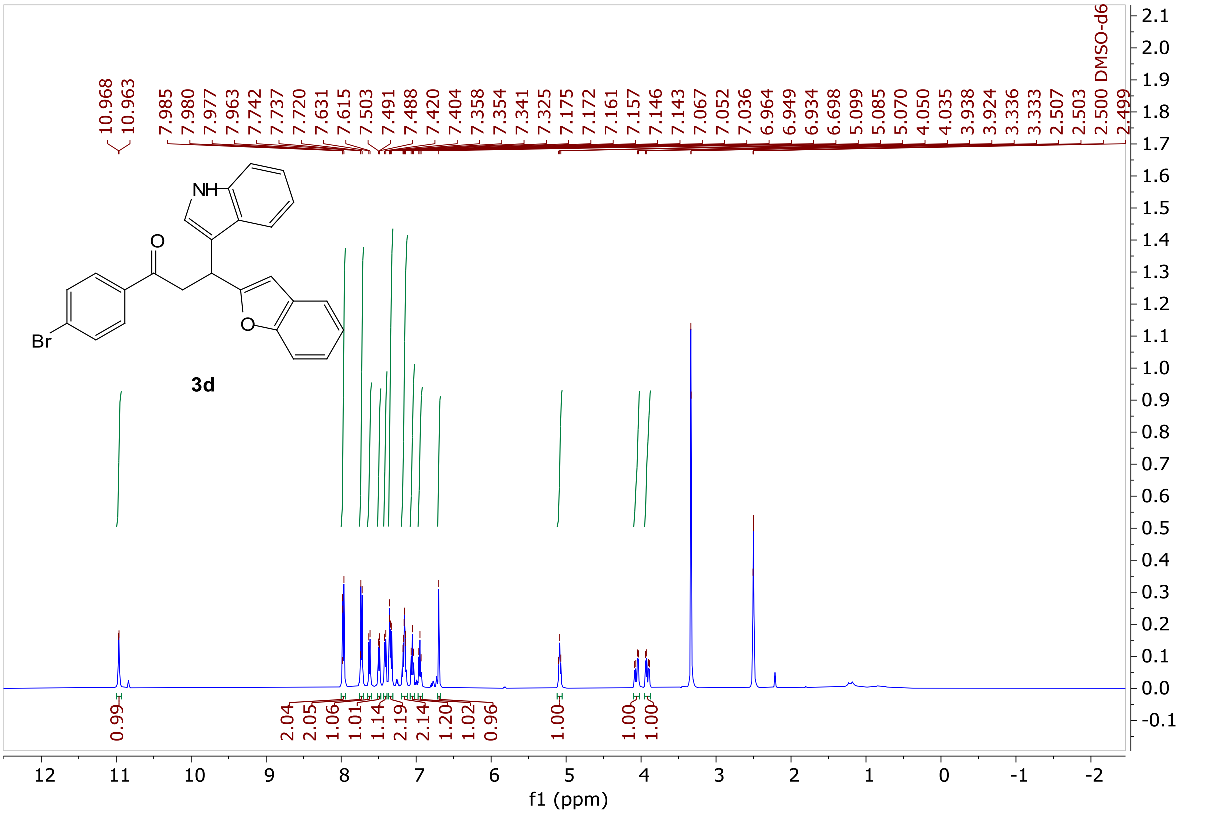

AB799FP_13C

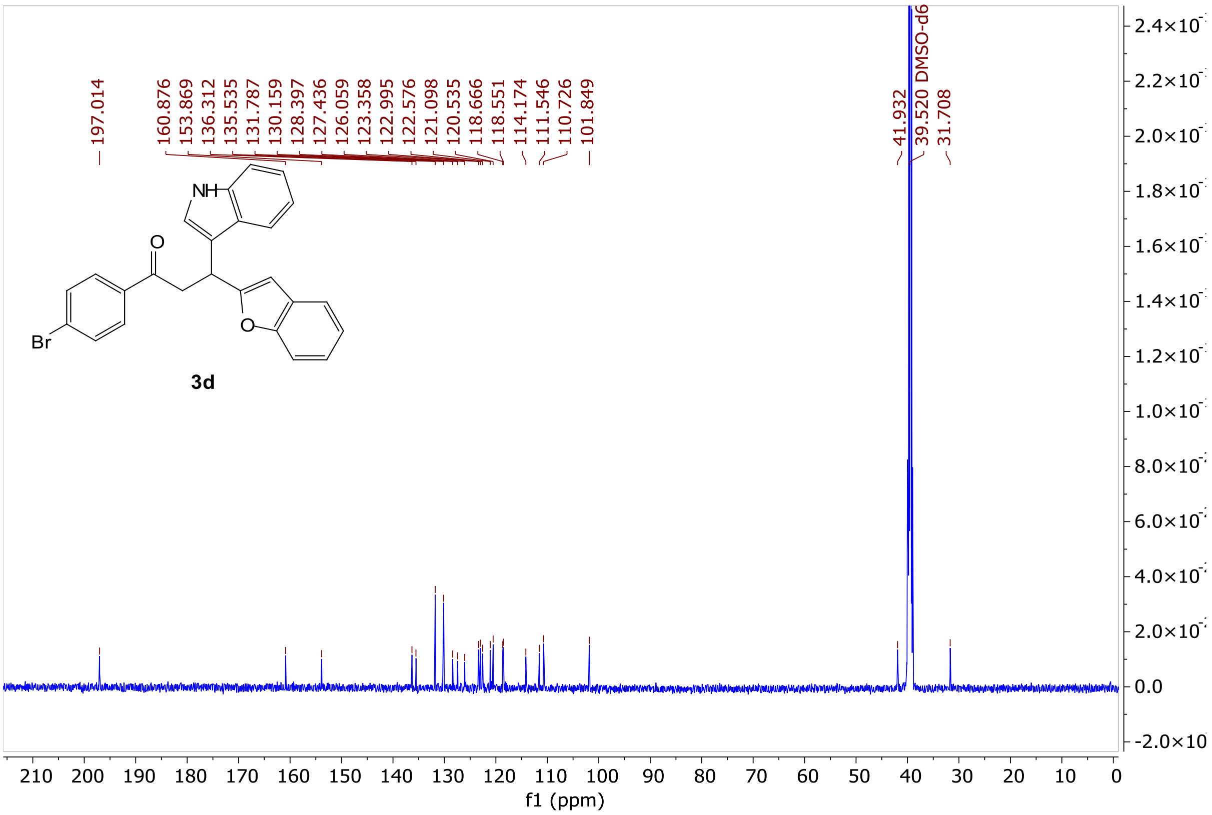

^{1}H-NMR and ^{13}C-NMR for Compound-3e

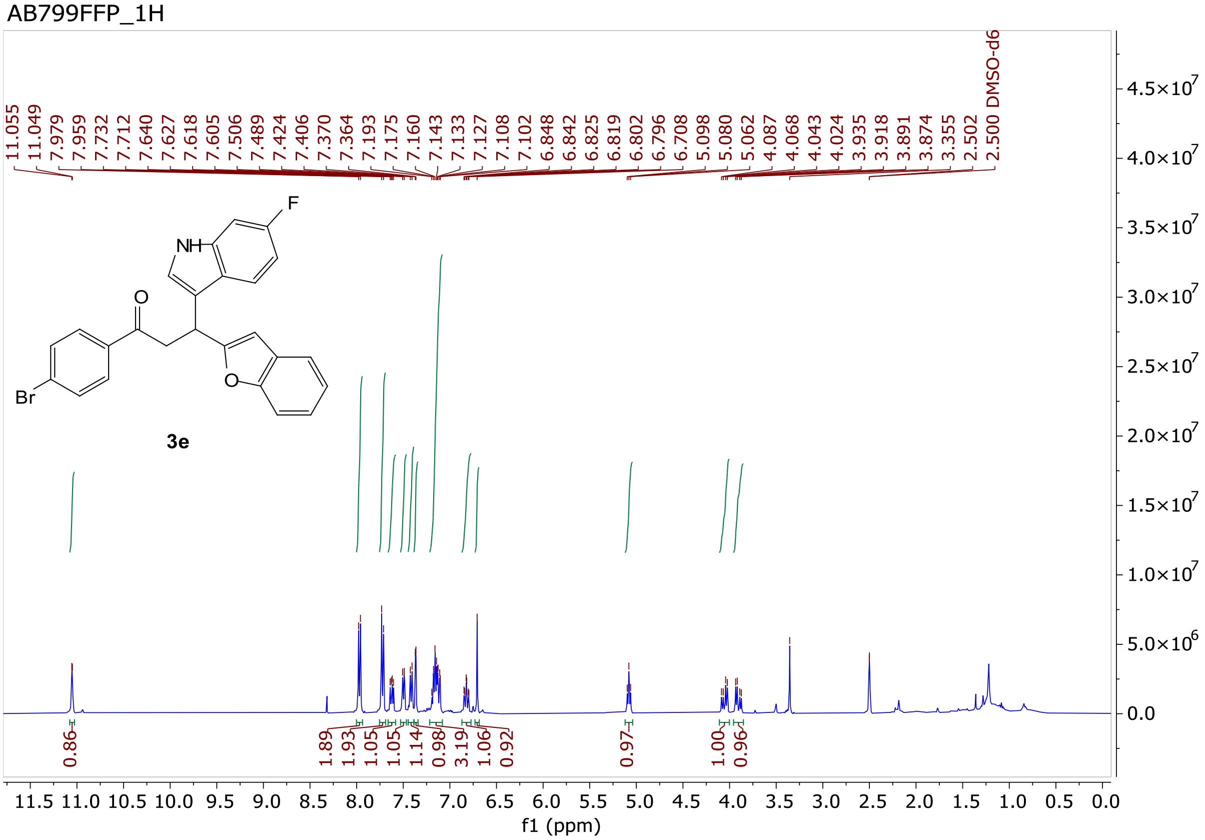

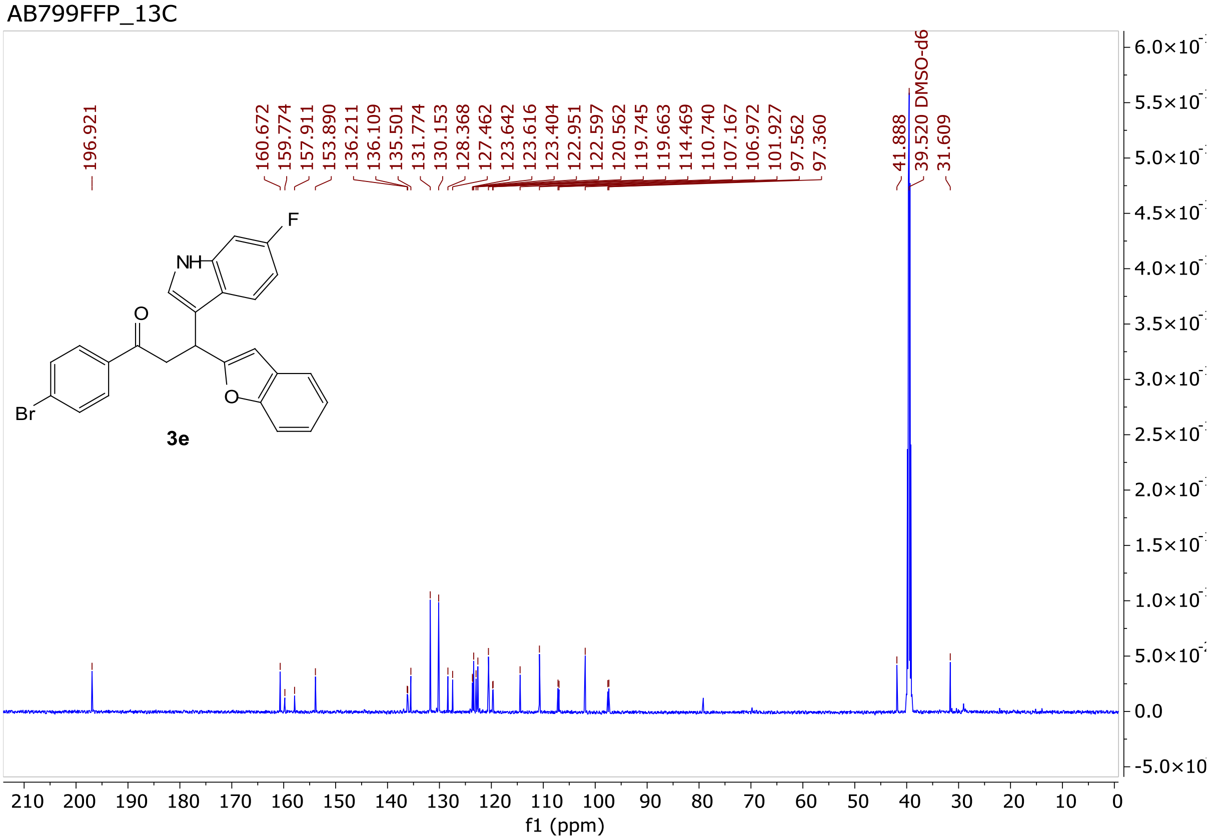

^{1}H-NMR and ^{13}C-NMR for compound-3f

AB812FP_1H

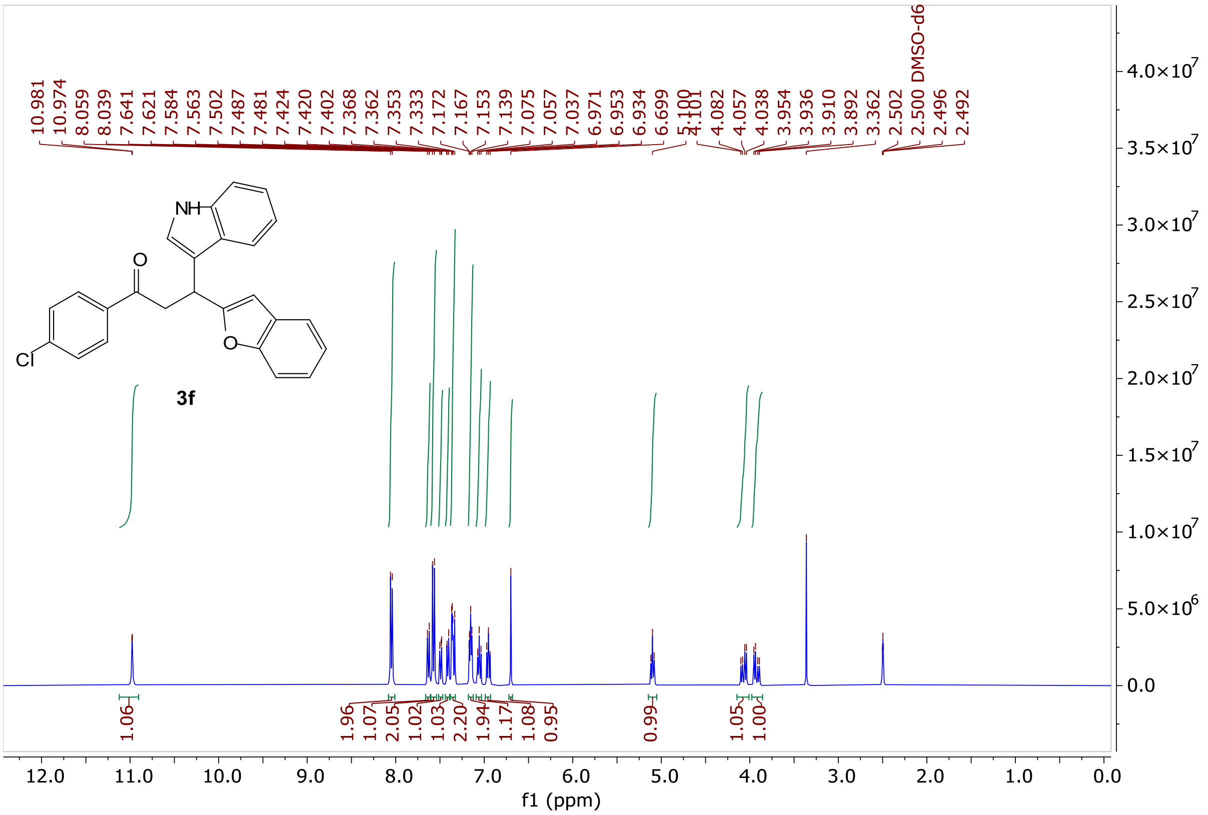

AB812FP_13C

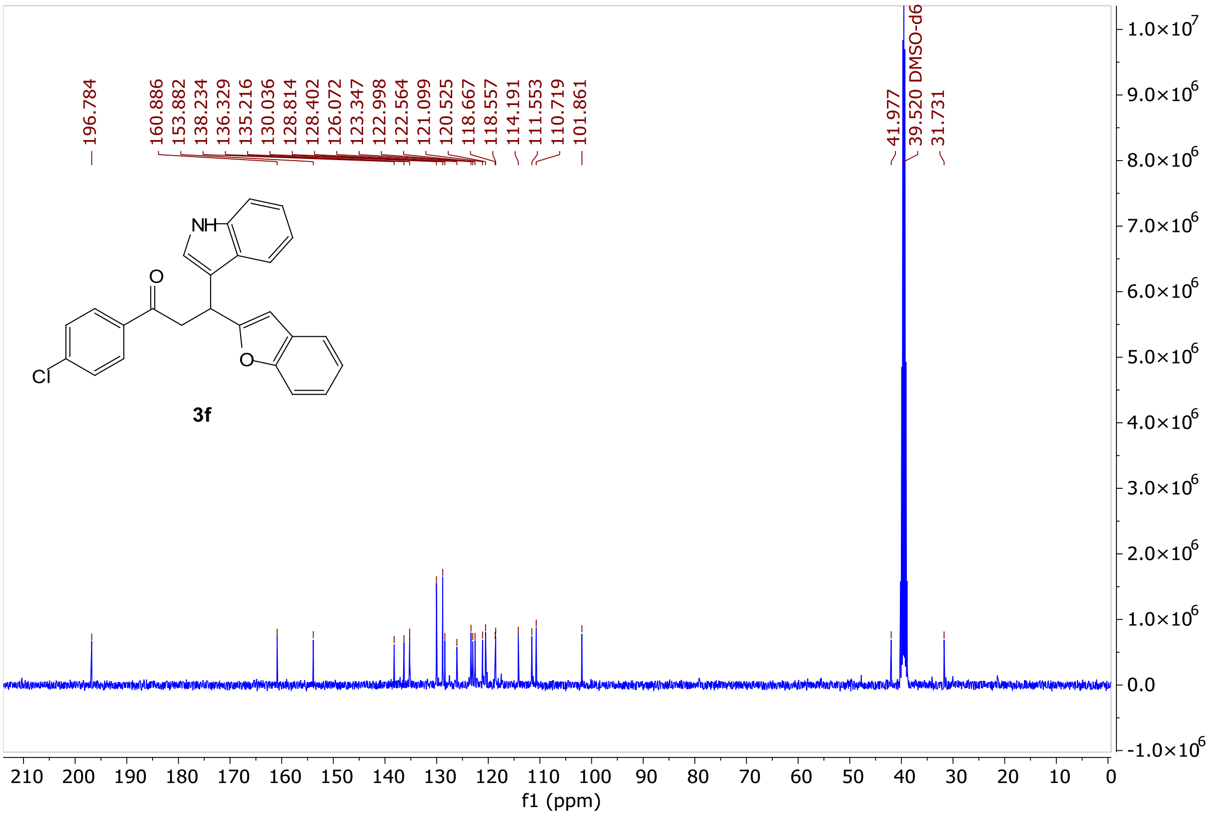

^{1}H-NMR and ^{13}C-NMR for compound-3g

AB812FFP_1H

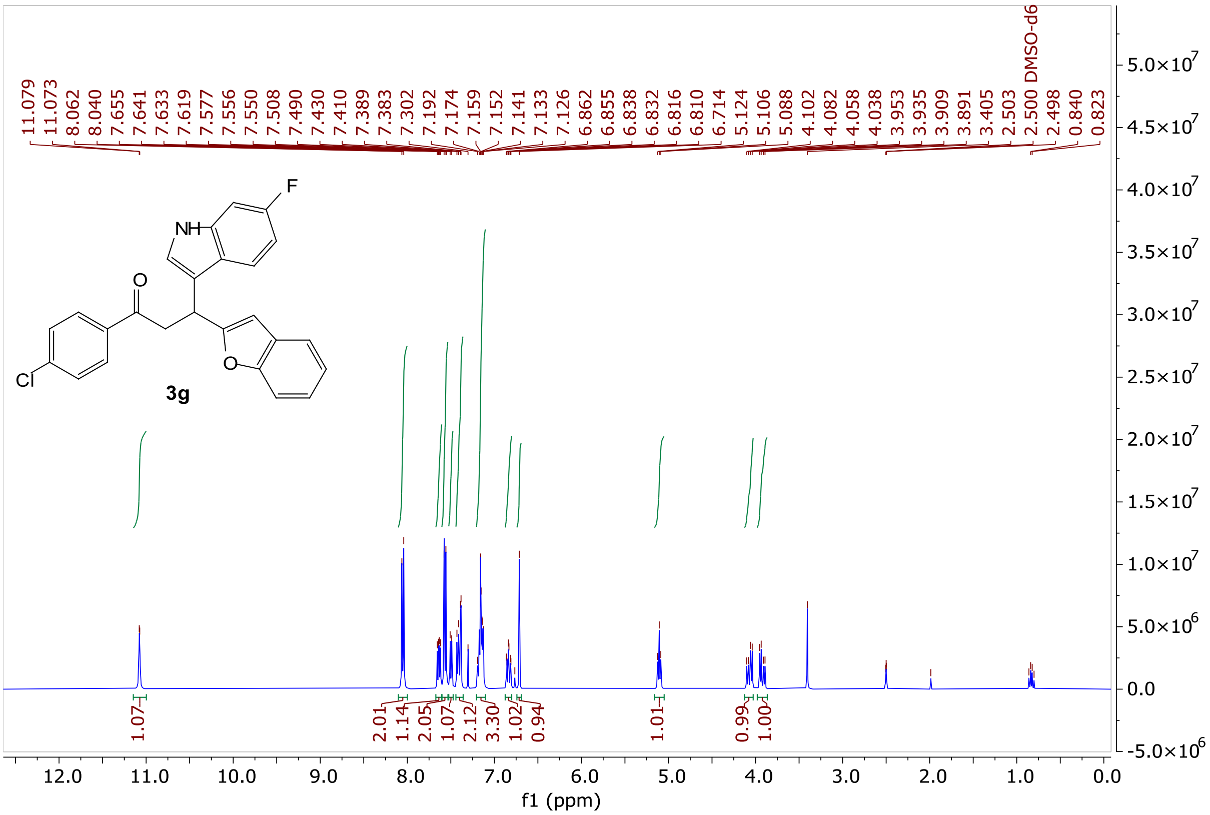

AB812FFP_13C

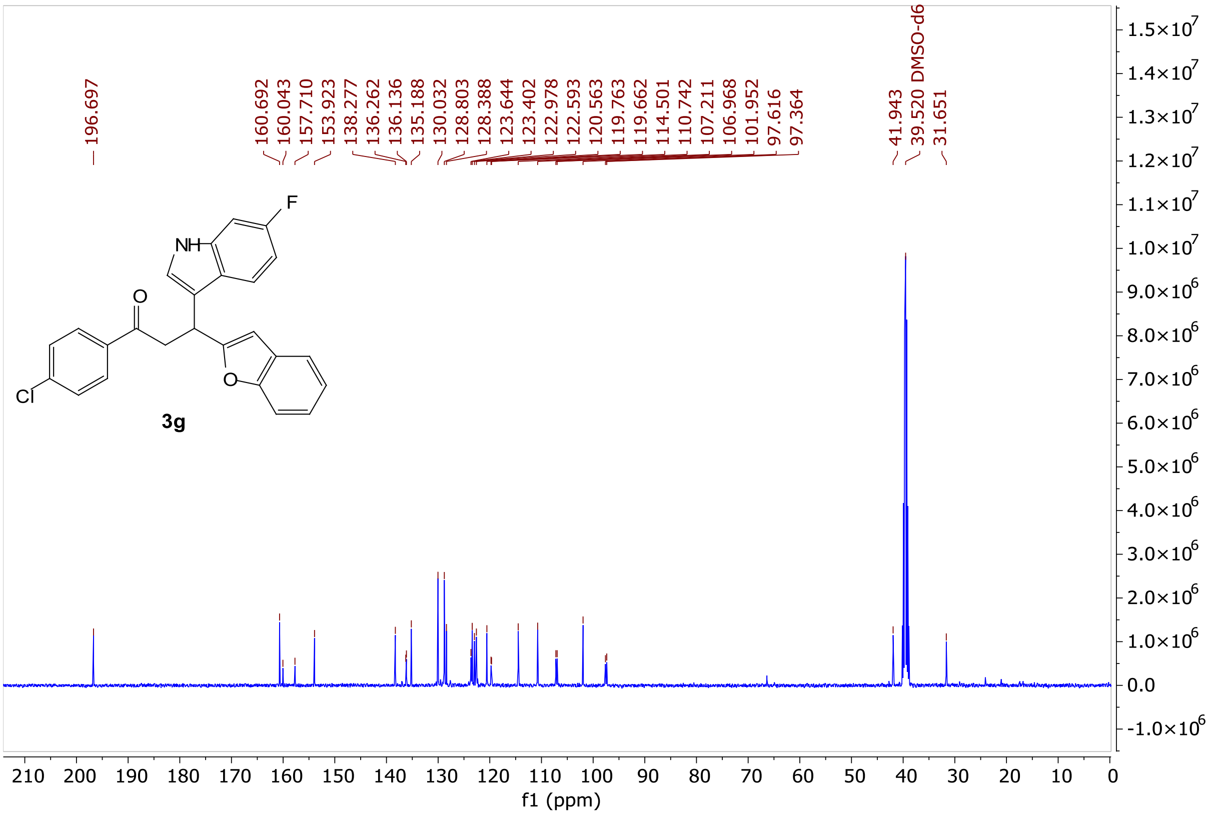

^{1}H-NMR and ^{13}C-NMR for compound-3h

AB864FP_1H

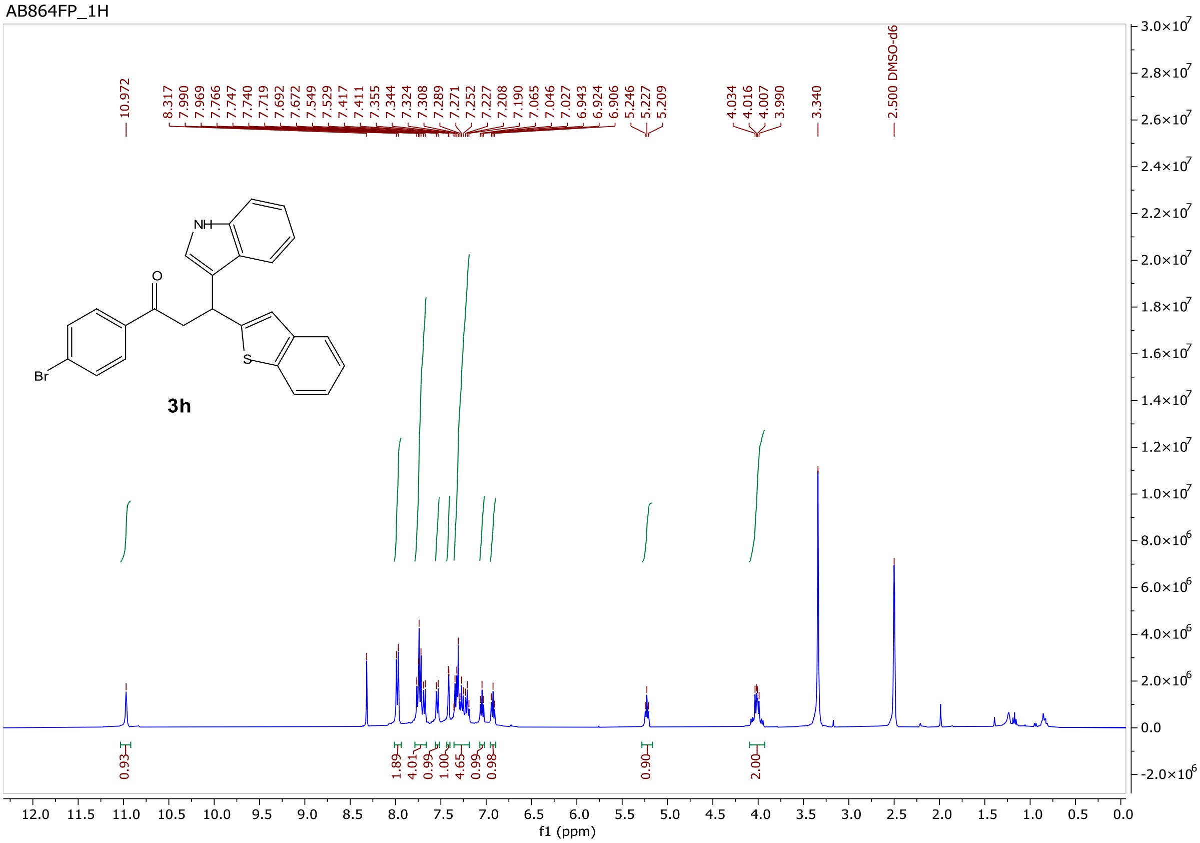

AB864_13C

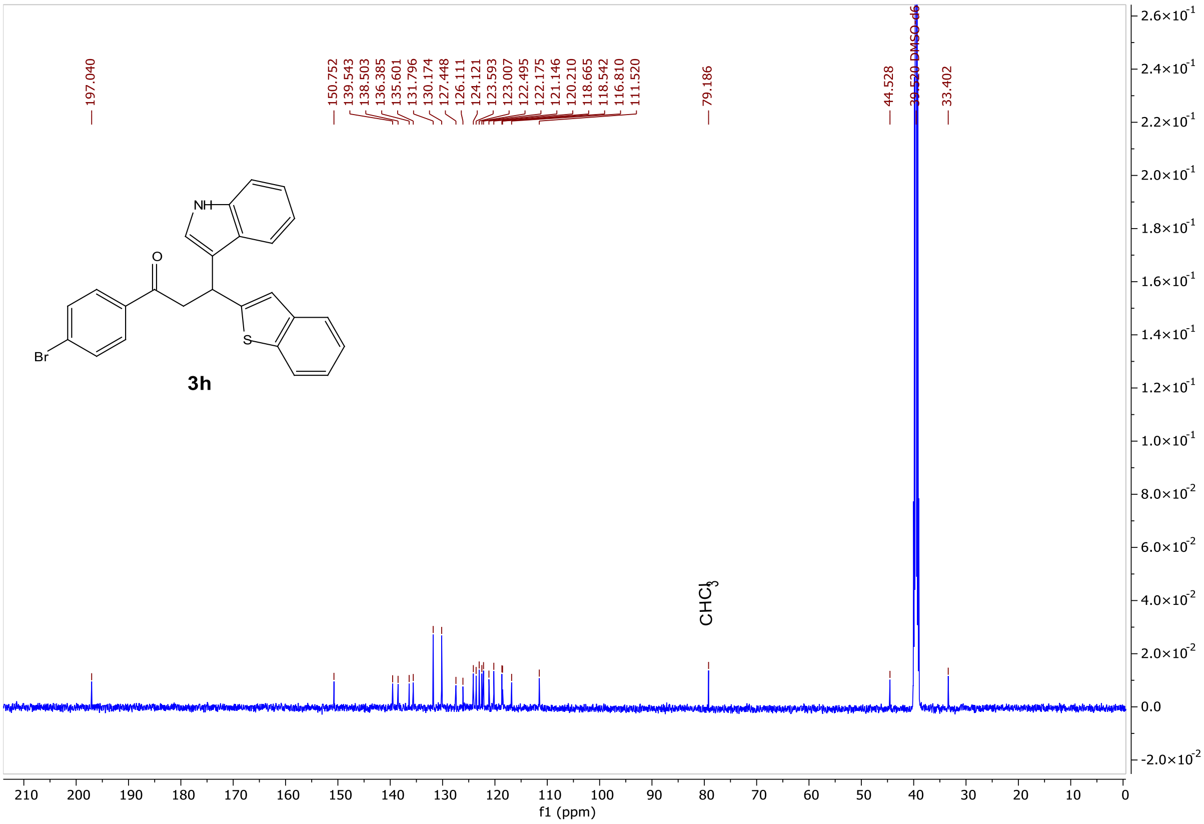

^{1}H-NMR and ^{13}C-NMR for compound-3i

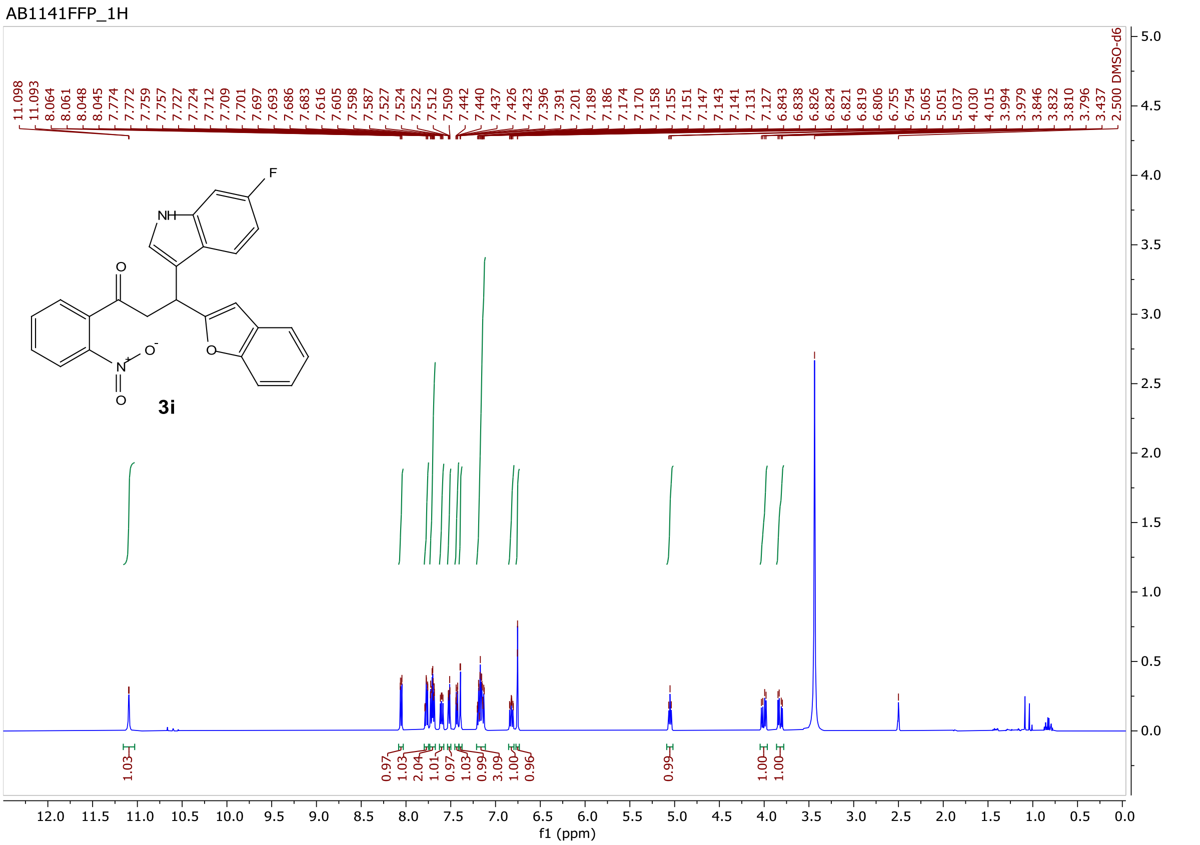

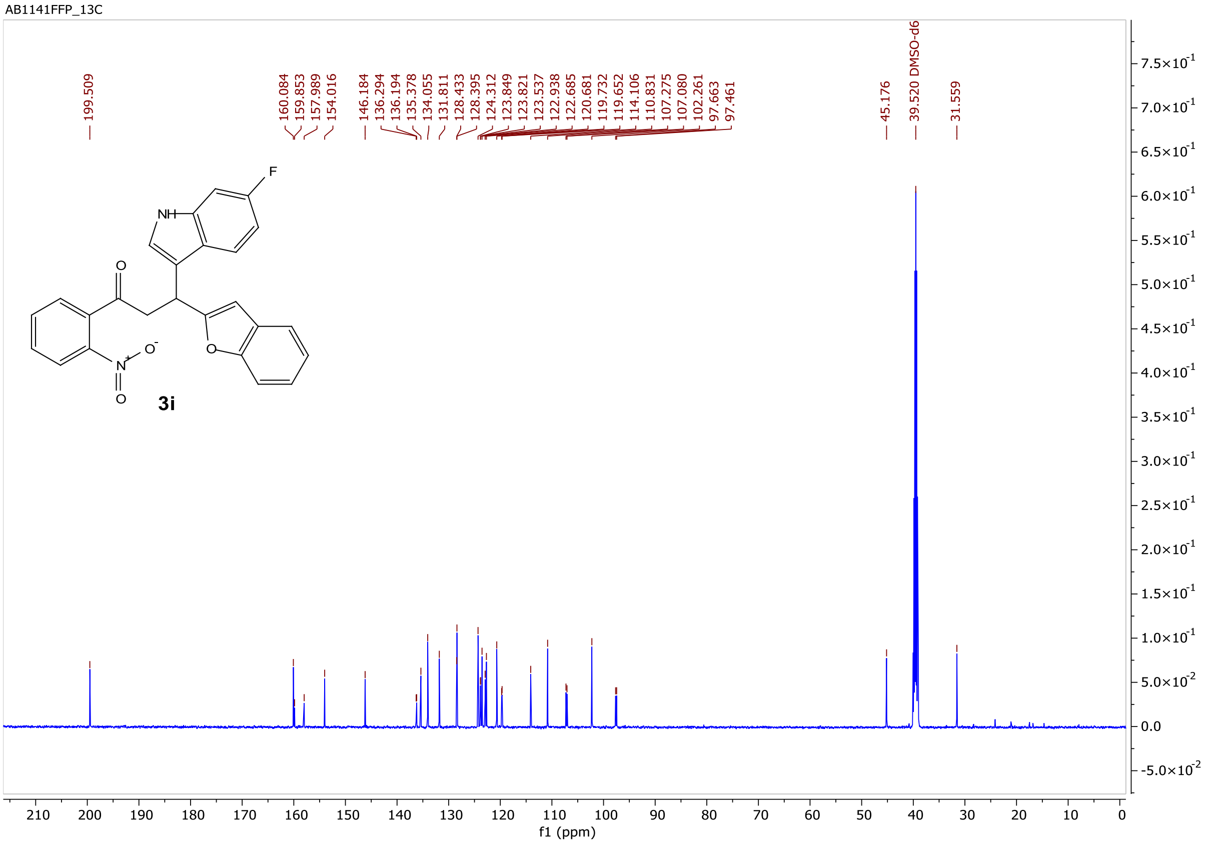

^{1}H-NMR and ^{13}C-NMR for compound-3j

AB1141BrFP_1H

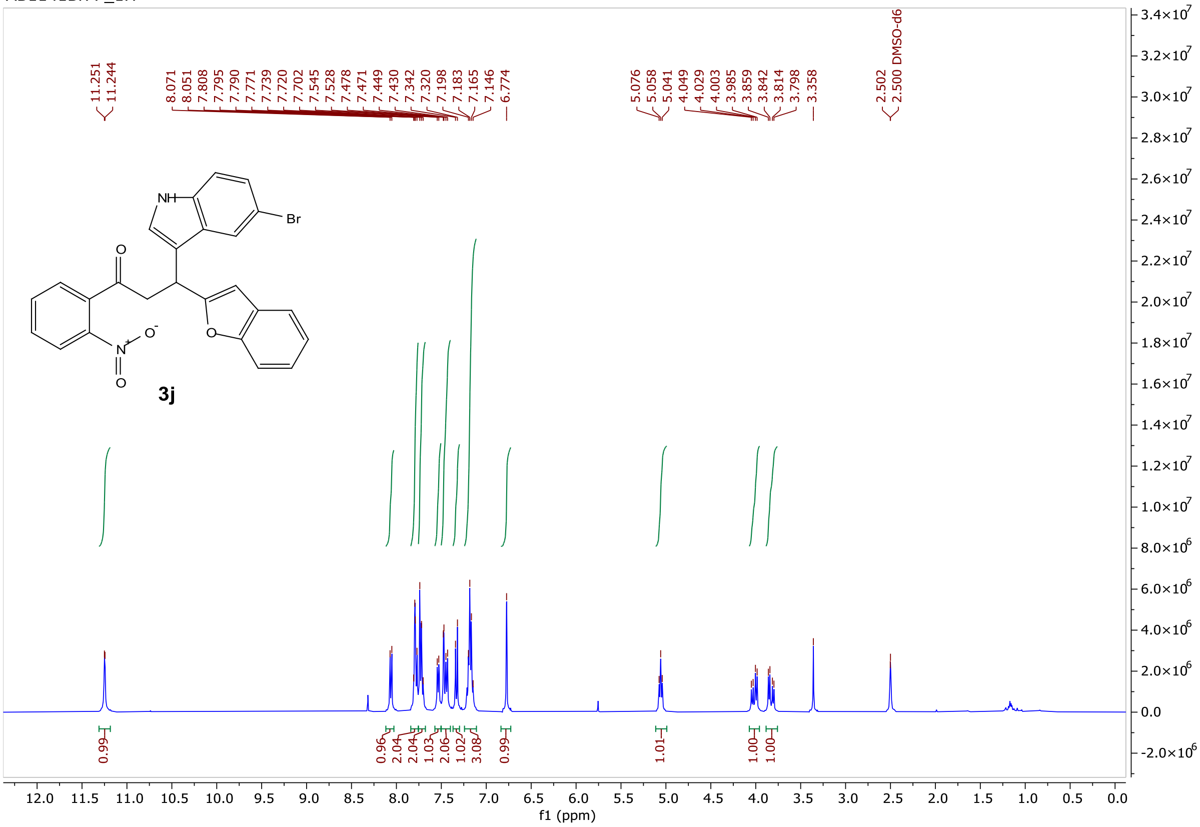

AB1141BrFP_13C

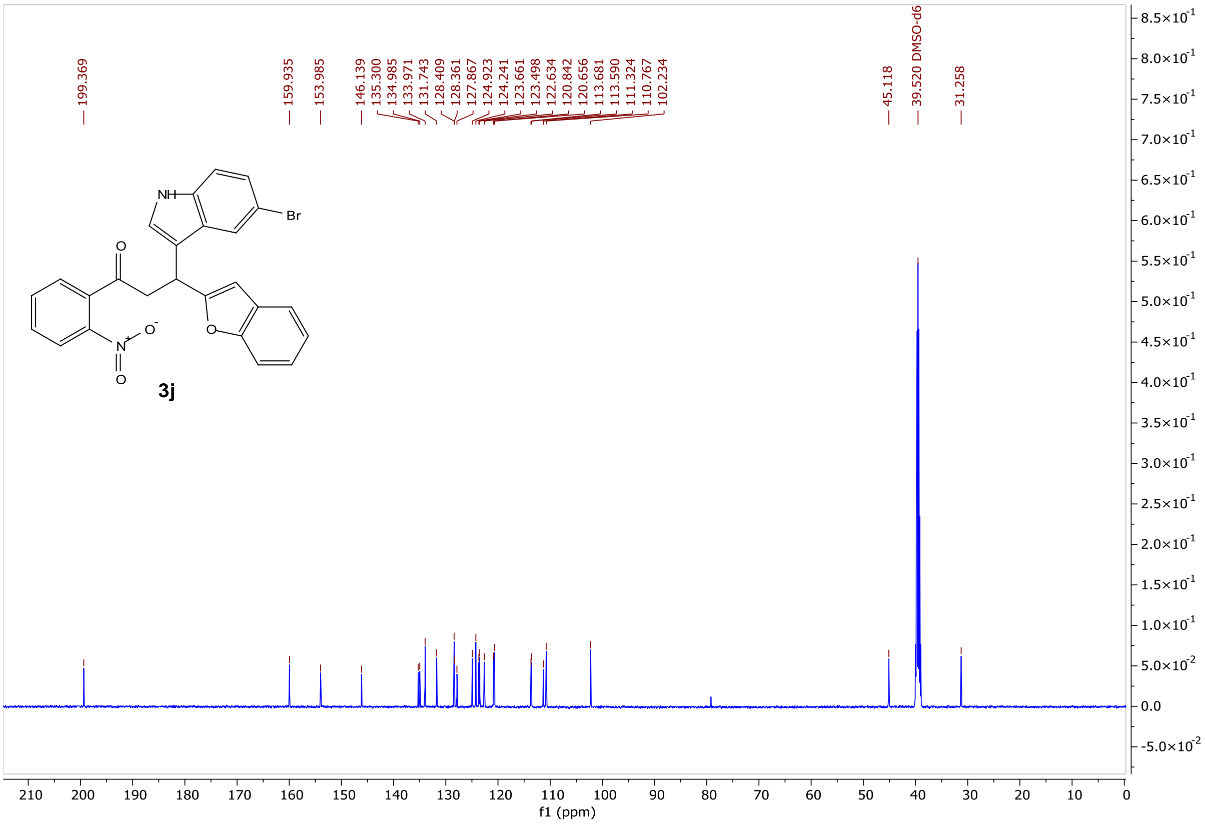

^{1}H-NMR and ^{13}C-NMR for compound-3k

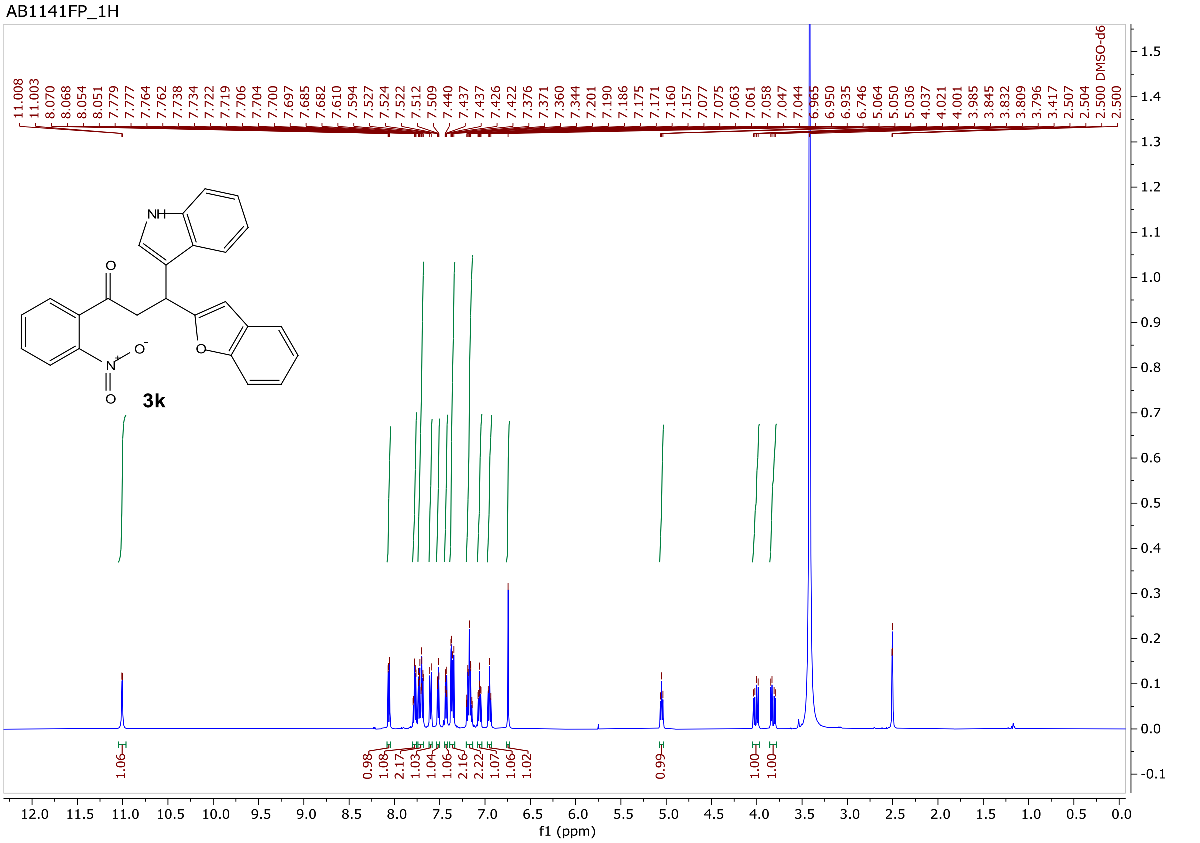

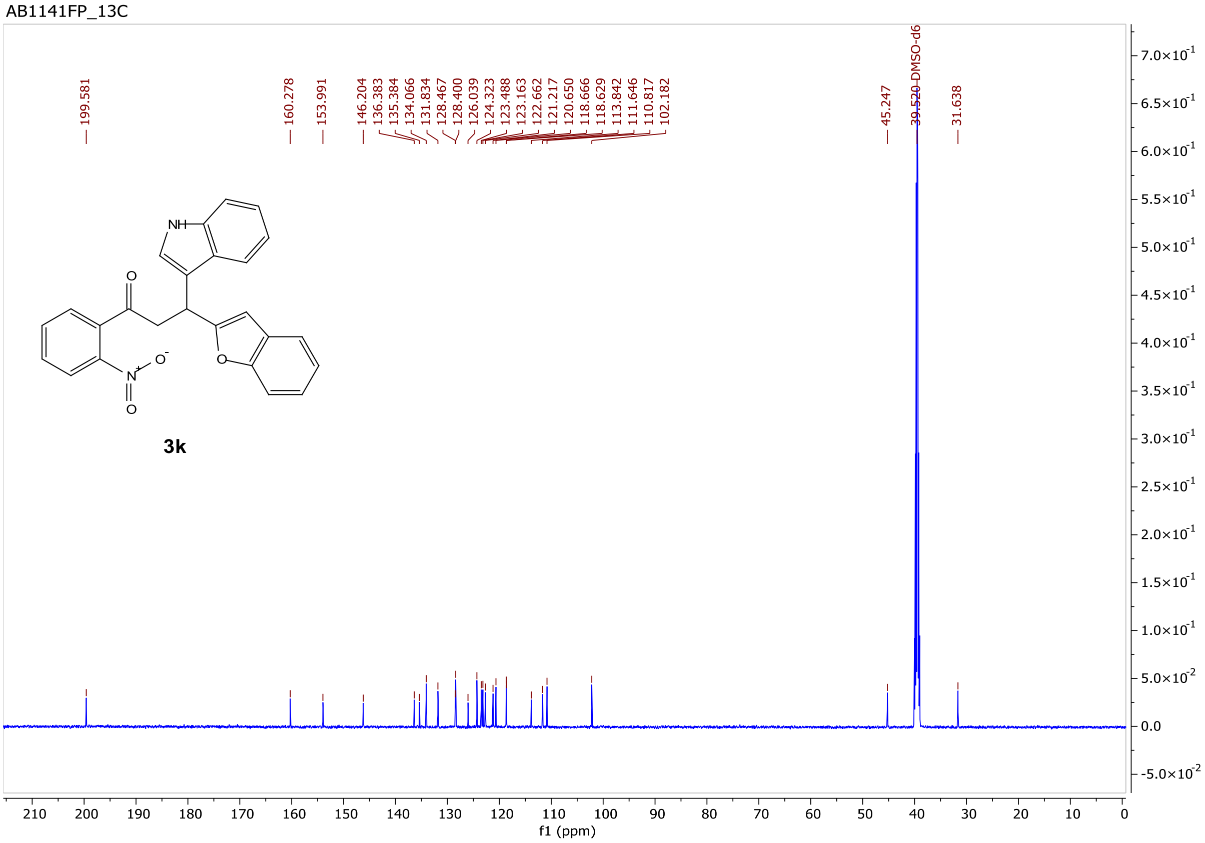

^{1}H-NMR and ^{13}C-NMR for compound-3l

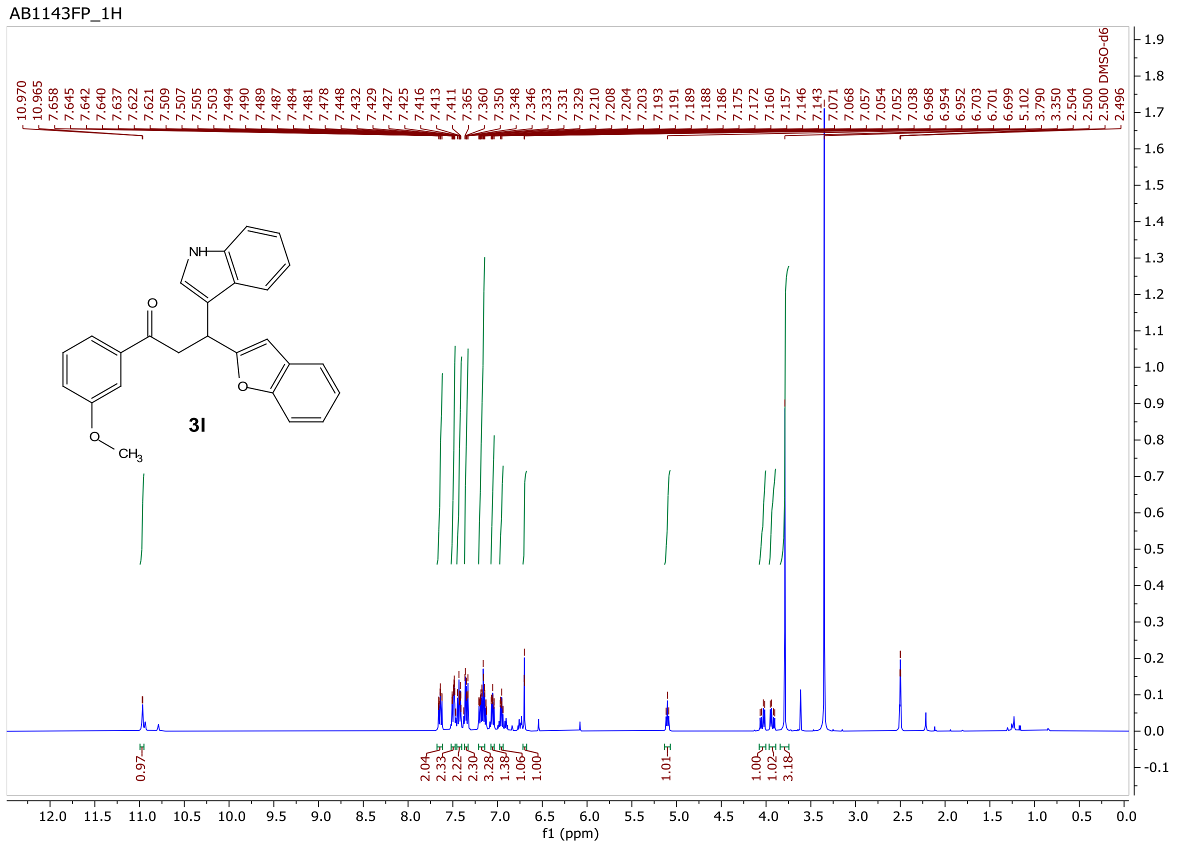

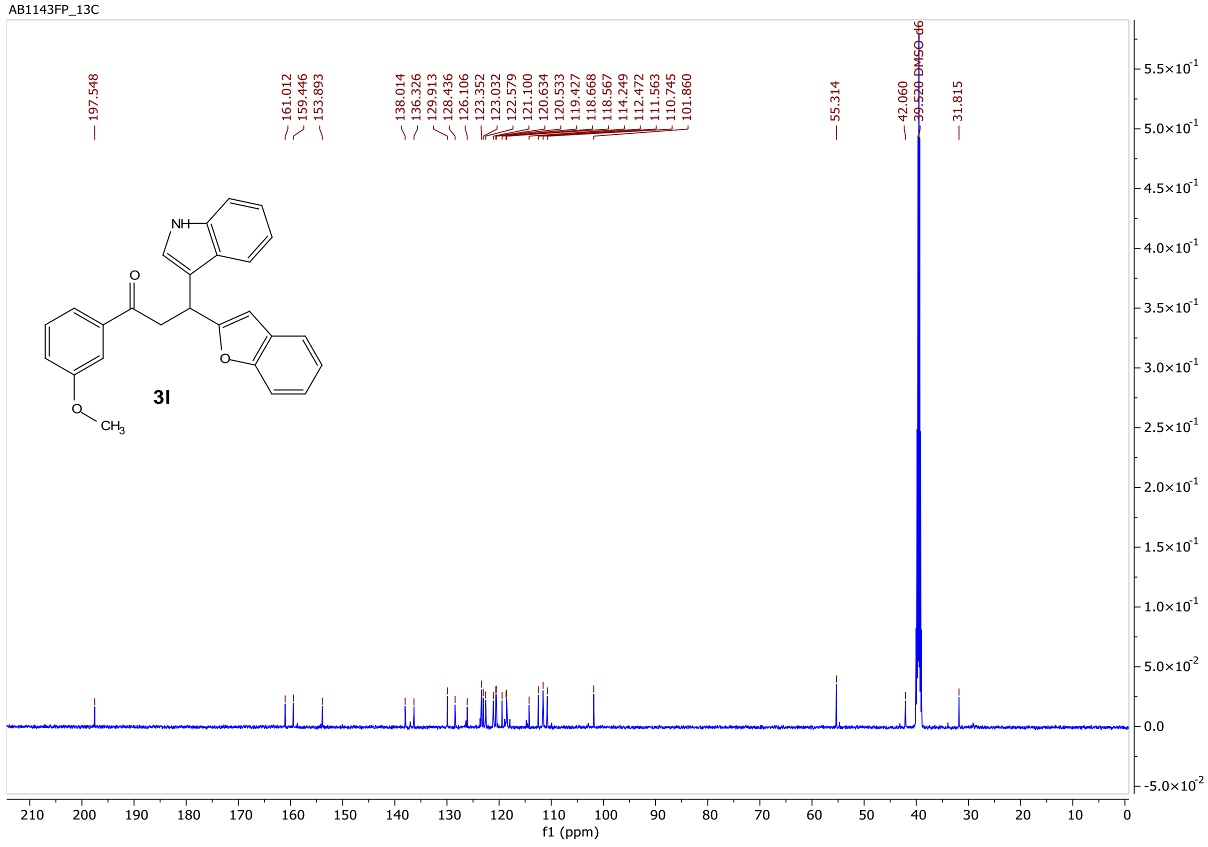

^{1}H-NMR and ^{13}C-NMR for compound-3m

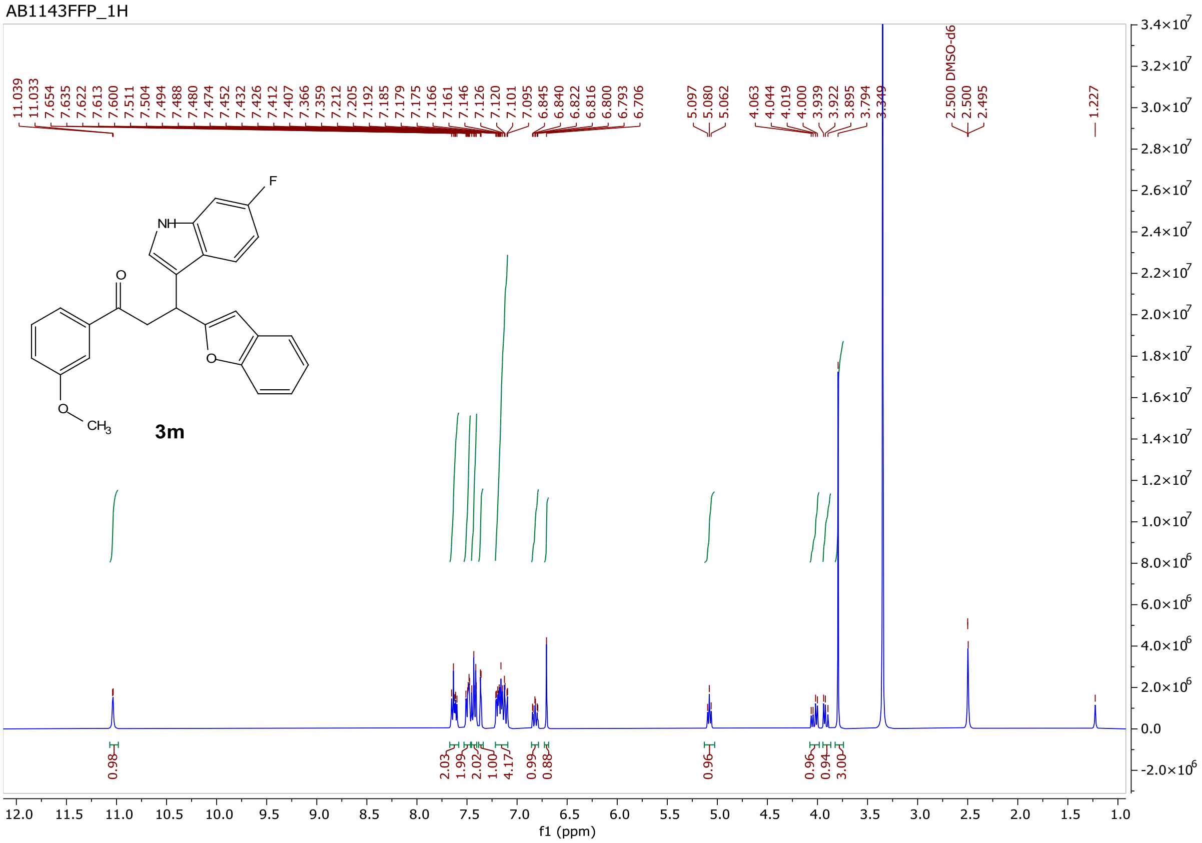

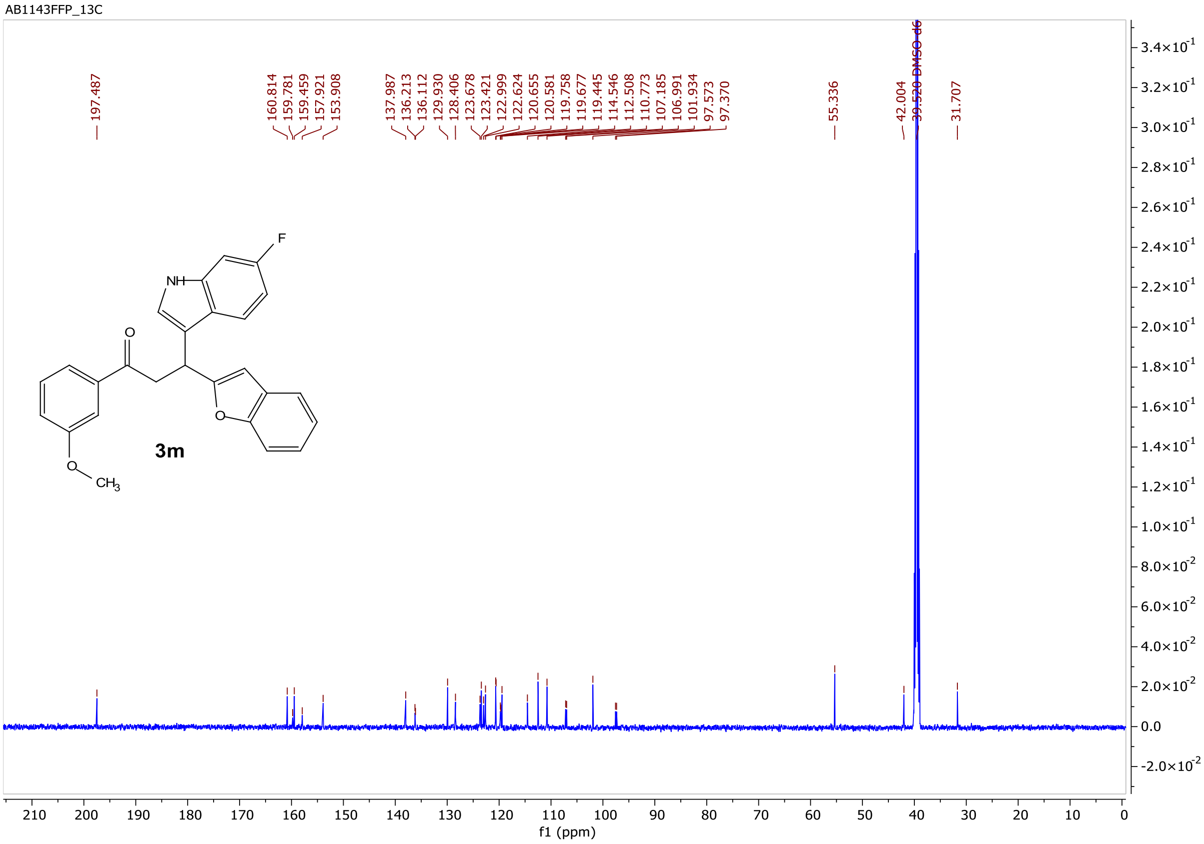

^{1}H-NMR and ^{13}C-NMR for compound-3n

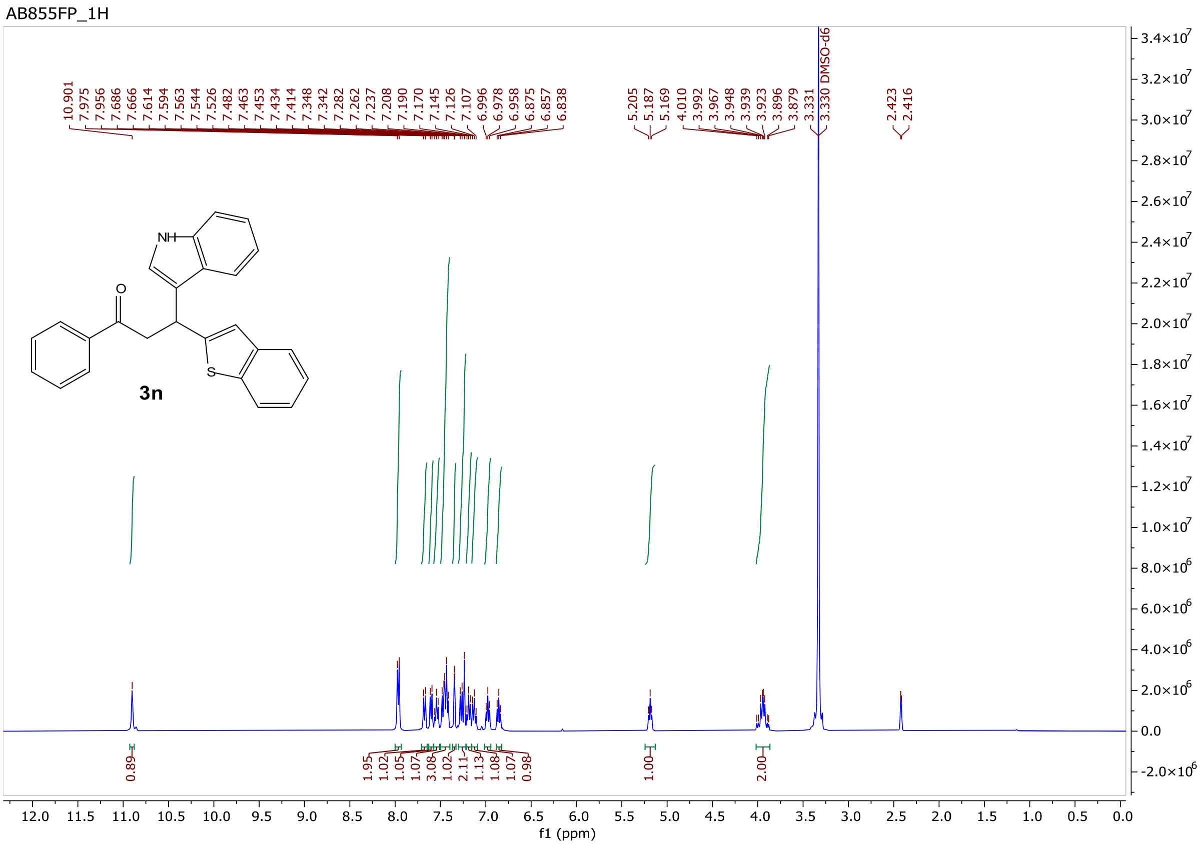

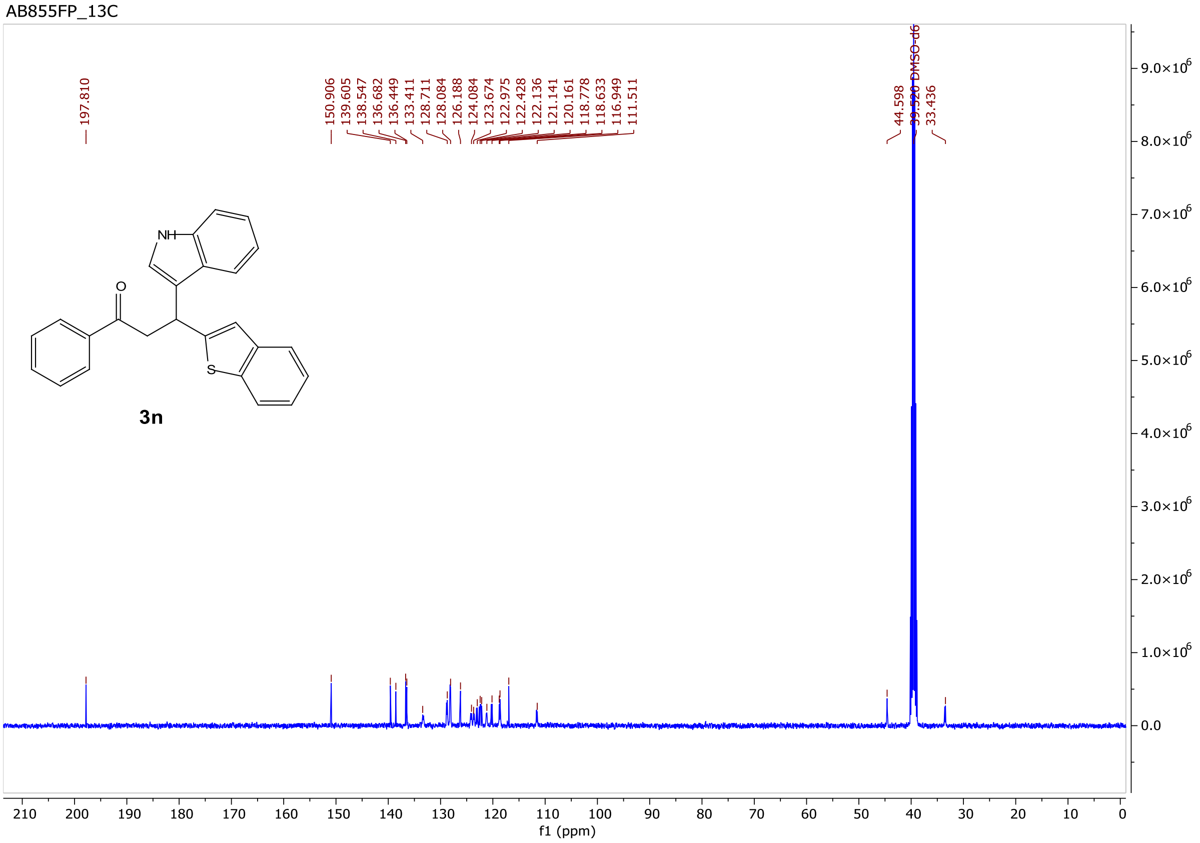

^{1}H-NMR and ^{13}C-NMR for compound-3o

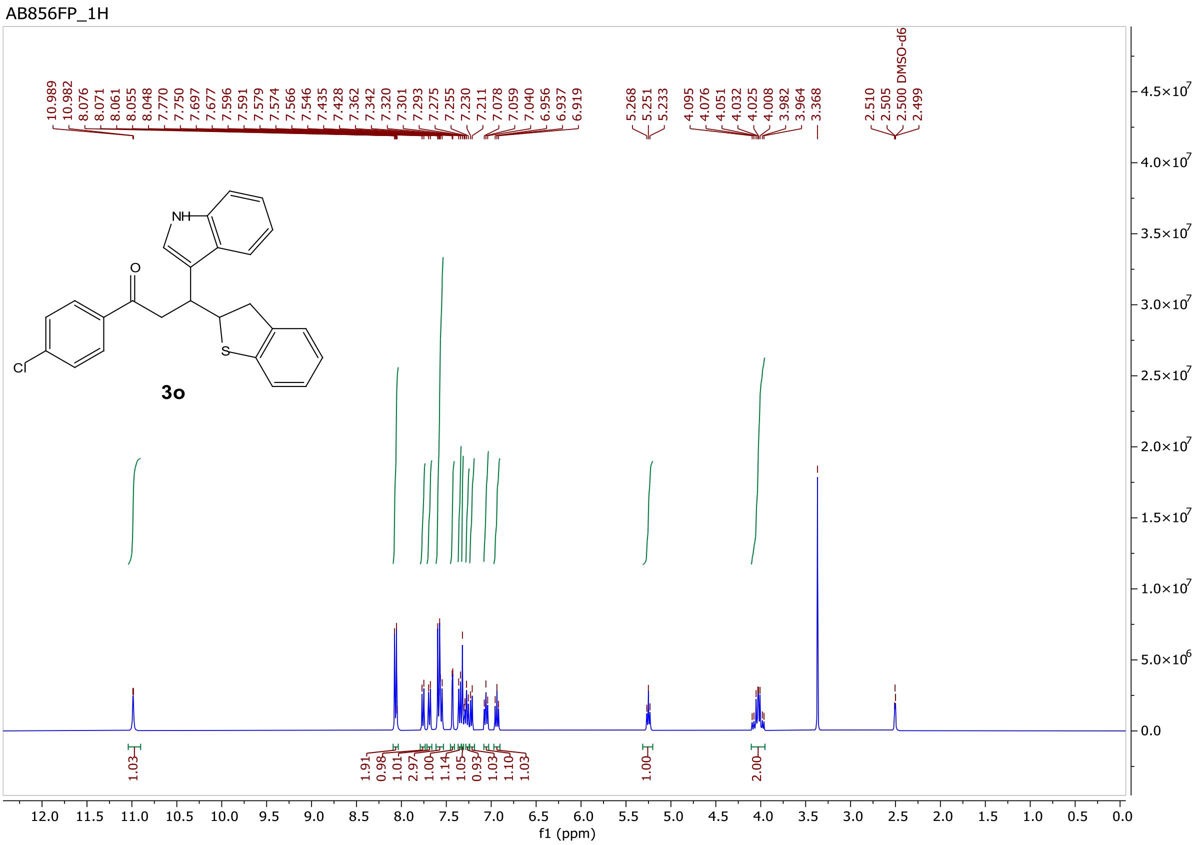

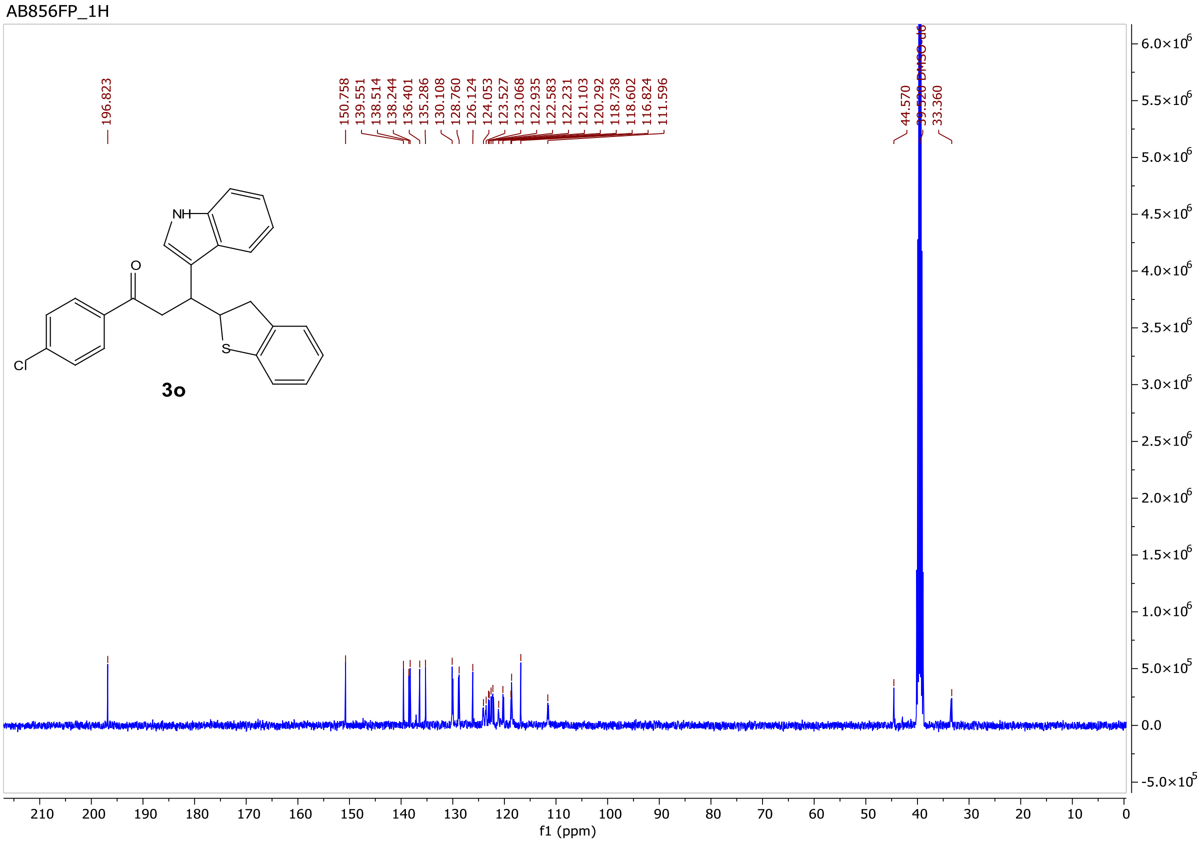

^{1}H-NMR and ^{13}C-NMR for compound-3p

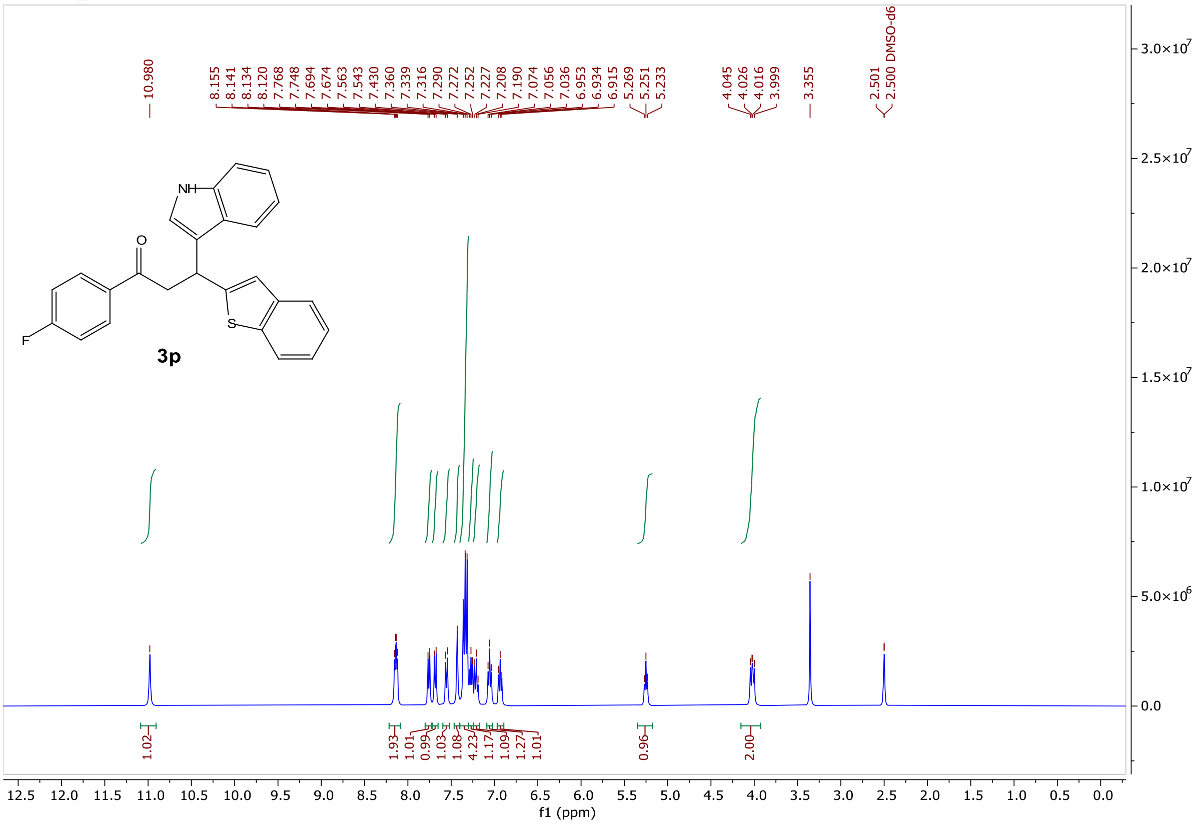

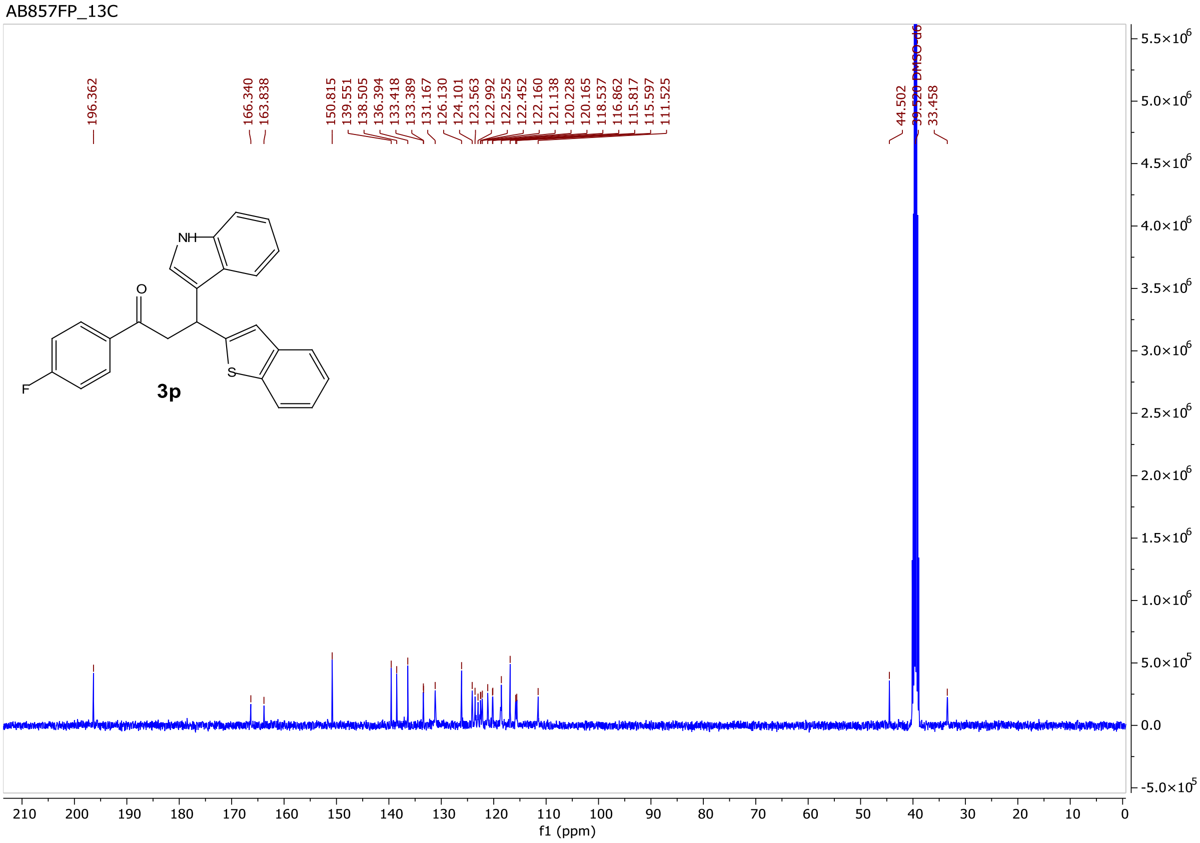

^{1}H-NMR and ^{13}C-NMR for compound-3q

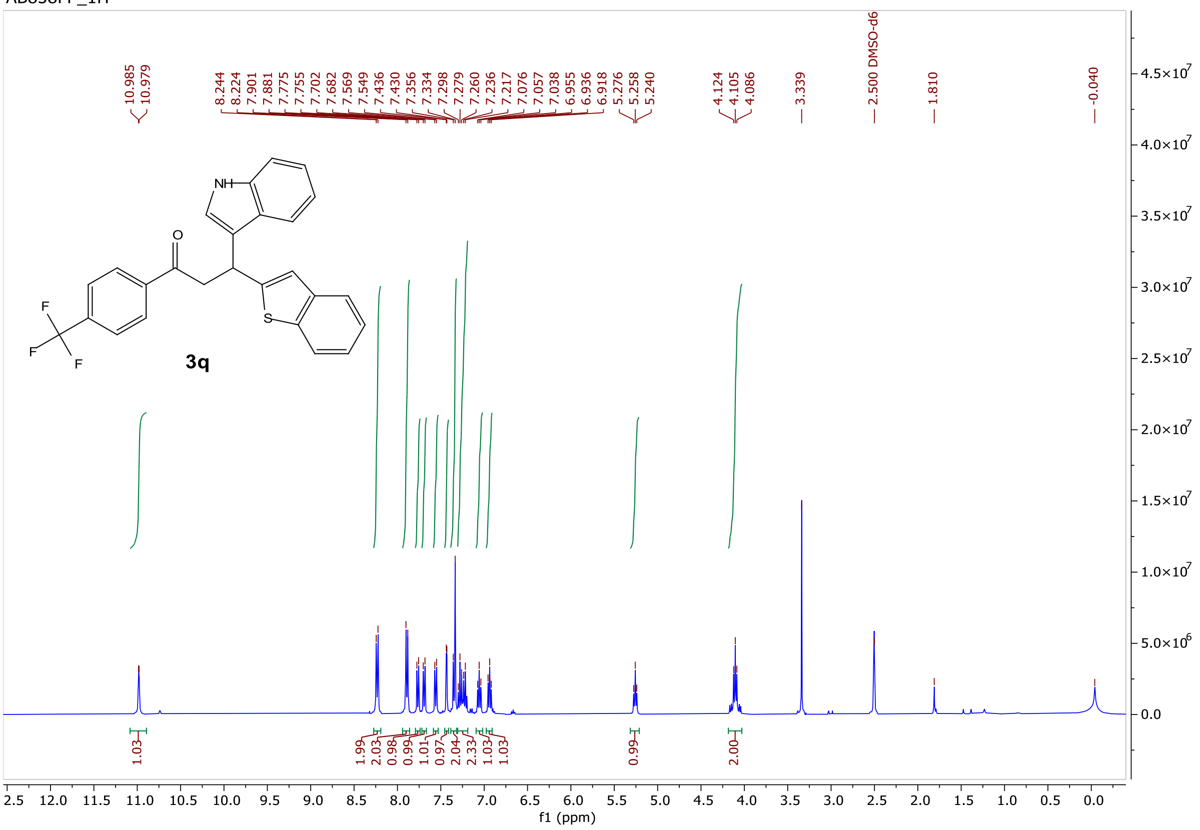

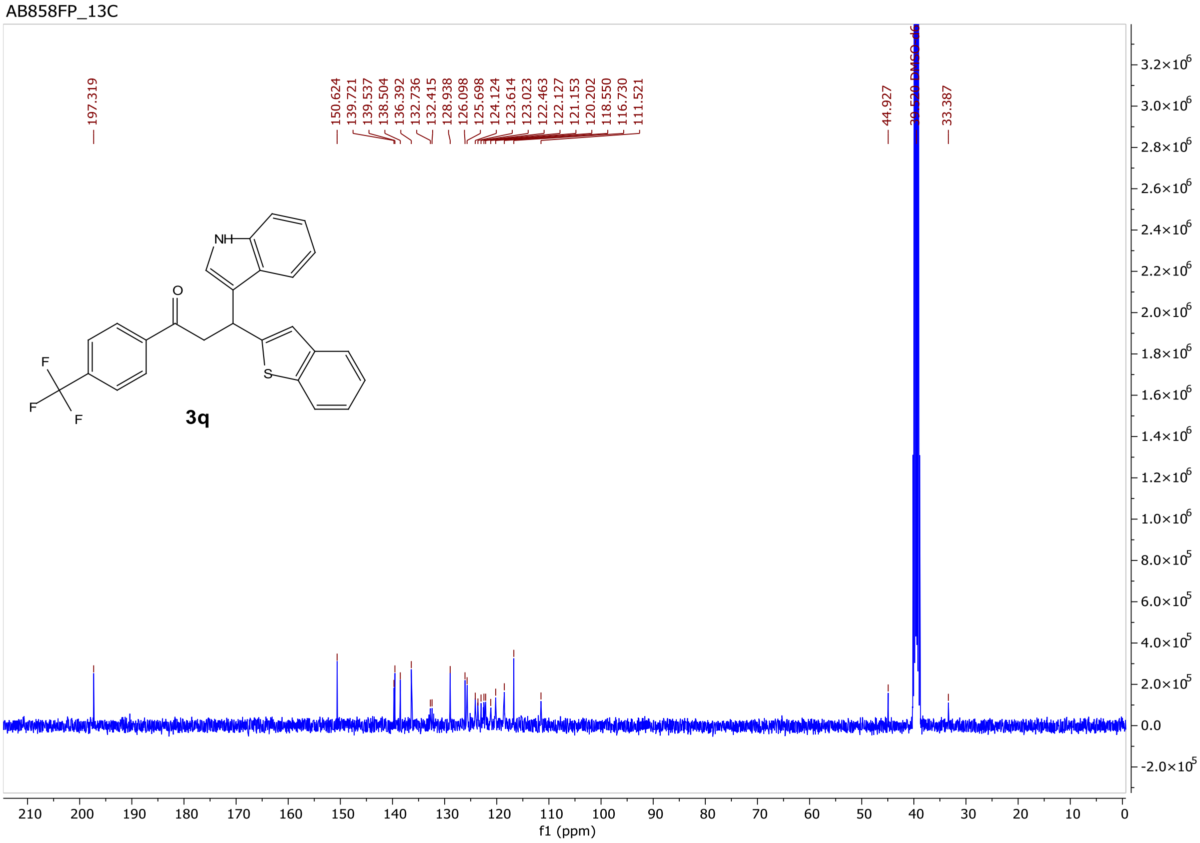

^{1}H-NMR and ^{13}C-NMR for compound-3r

AB1144FP_1H

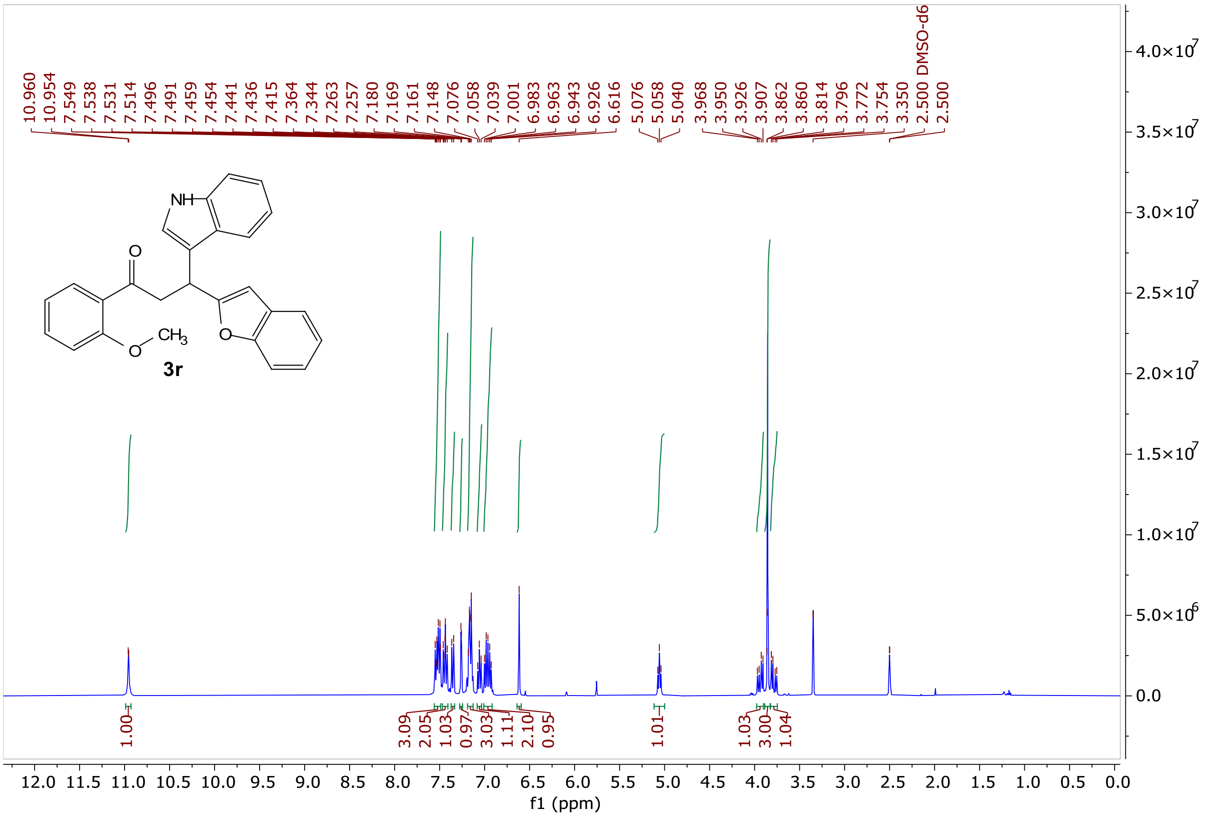

AB1144FP_13C

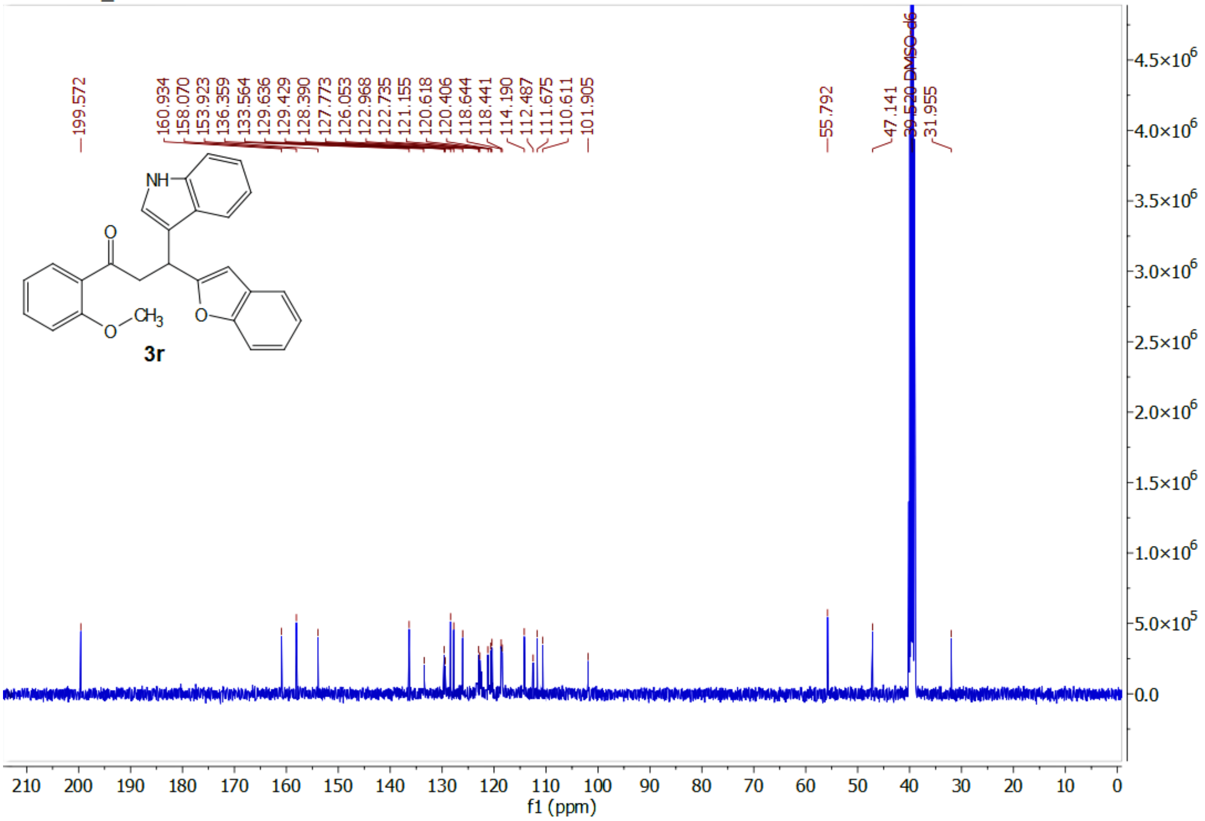

^{1}H-NMR and ^{13}C-NMR for compound-3s

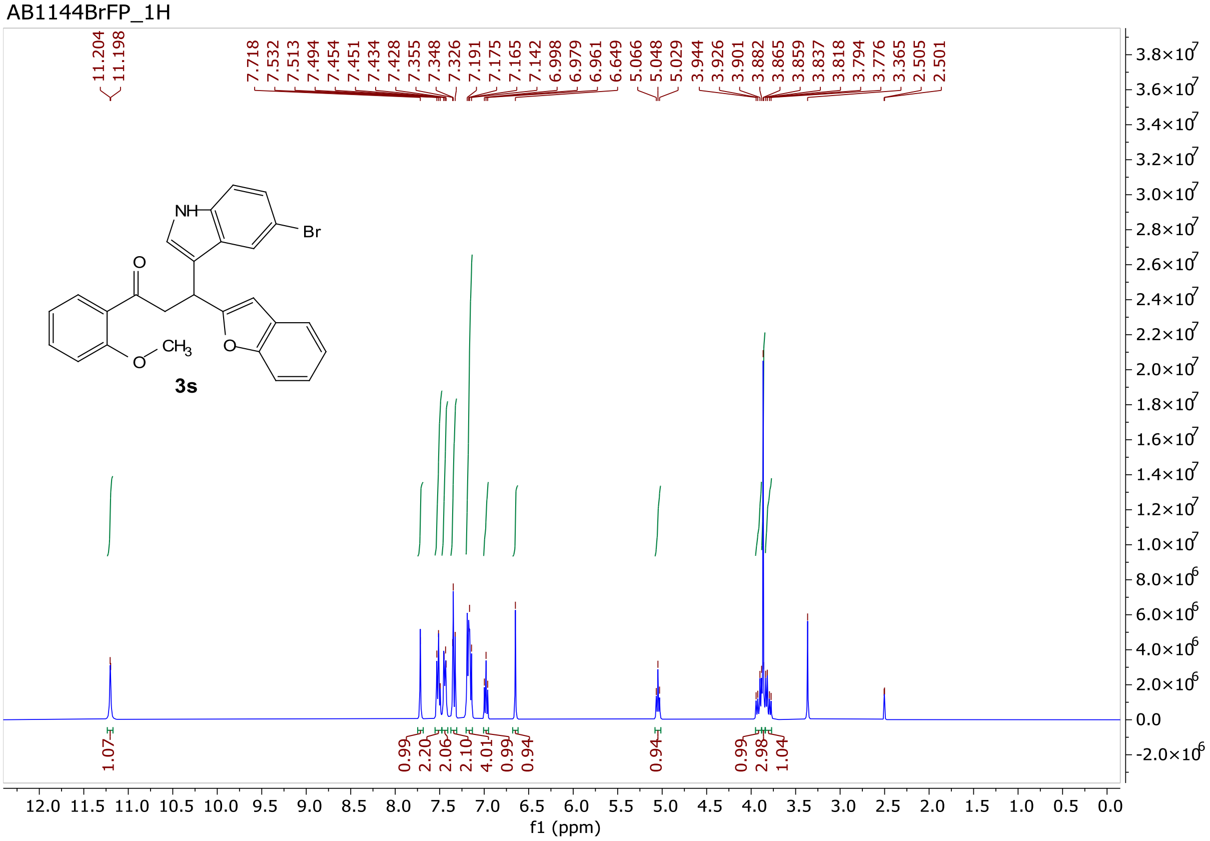

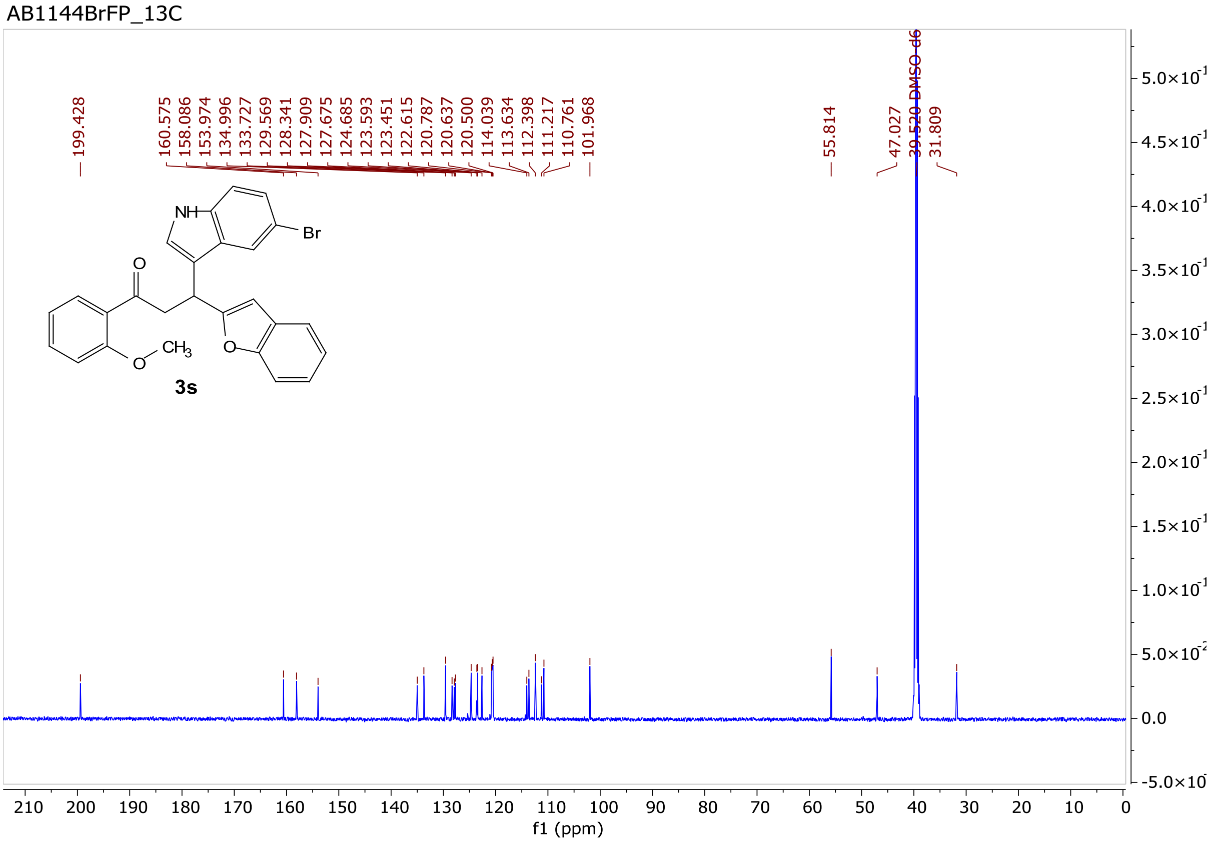

The Biological Activity Assays Protocols

A. PC3 cells (Prostate Cancer) assay

Cytotoxic activity of compounds was evaluated in 96-well flat-bottomed micro plates by using the standard MTT (3-[4, 5-dimethylthiazole-2-yl]-2, 5-diphenyl-tetrazolium bromide) colorimetric assay. For this purpose, PC3 cells (Prostate Cancer) were cultured in Dulbecco's Modified Eagle Medium, supplemented with 5% of fetal bovine serum (FBS), 100 IU/ml of penicillin and 100 μg/ml of streptomycin in 75 cm^2 flasks, and kept in 5% CO_2 incubator at 37°C. Exponentially growing cells were harvested, counted with haemocytometer and diluted with a particular medium. Cell culture with the concentration of $1x10^5$ cells/ml was prepared and introduced (100 μL/well) into 96-well plates. After overnight incubation, medium was removed and 200 μL of fresh medium was added with different concentrations of compounds (1-30μM). After 48 hrs, 200 μL MTT (0.5 mg/ml) was added to each well and incubated further for 4 hrs. Subsequently, 100μL of DMSO was added to each well. The extent of MTT reduction to formazan within cells was calculated by measuring the absorbance at 570 nm, using a micro plate reader (Spectra Max plus, Molecular Devices, CA, USA). The cytotoxicity was recorded as concentration causing 50% growth inhibition (IC_{50}) for PC3 cells. The percent inhibition was calculated by using the following formula:

% inhibition = 100-((mean of O.D of test compound – mean of O.D of negative control)/ (mean of O.D of positive control – mean of O.D of negative control)*100).

The results (% inhibition) were processed by using Soft- Max Pro software (Molecular Device, USA).

STANDARD DRUG:

Standard drug used in the MTT assay was doxorubicin.

ATCC #: CRL-1435 (Lot. No. 58501591)

Reference:

1. Mosmann T. Rapid colorimetric assay for cellular growth and survival: application to proliferation and cytotoxicity assays. *J Immunol Meth* **1983**, *65*, 55–63.

B. Cytotoxicity assay Protocol (BJ *Human fibroblast cells*)

Cytotoxic activity of compounds was evaluated in 96-well flat-bottomed micro plates by using the standard MTT (3-[4, 5-dimethylthiazole-2-yl]-2, 5-diphenyl-tetrazolium bromide) colorimetric assay (Price & J. McMillan1, 1990). For this purpose, BJ *Human fibroblast* cells were cultured in Dulbecco's Modified Eagle Medium, supplemented with 10% of fetal bovine serum (FBS), 100 IU/ml of penicillin and 100 μg/ml of streptomycin and 2 mM L-glutamine in 75 cm^2 flasks, and kept in 5% CO_2 incubator at 37°C (Mannerström, Toimela, Sarkanen & Heinonen, 2017).

Exponentially growing cells were harvested, counted with haemocytometer and diluted with a particular medium. Cell culture with the concentration of $6x10^4$cells/ml was prepared and introduced (100 μL/well) into 96-well plates. After overnight incubation, medium was removed and 200 μL of fresh medium was added with different concentrations of compounds (1-30μM). After 48 hrs, 200 μL MTT (0.5 mg/ml) was added to each well and incubated further for 3 hrs.

Subsequently, 100μL of DMSO was added to each well. The extent of MTT reduction to formazan within cells was calculated by measuring the absorbance at 550 nm, using a micro plate reader (Spectra Max plus, Molecular Devices, CA, USA). The cytotoxicity was recorded as concentration causing 50% growth inhibition (IC_{50}) for BJ cells. The percent inhibition was calculated by using the following formula:

% inhibition = 100-((mean of O.D of test compound – mean of O.D of negative control)/ (mean of O.D of positive control – mean of O.D of negative control)*100).

The results (% inhibition) were processed by using Soft- Max Pro software (Molecular Device, USA).

Following concentrations were used to calculate (in case) of IC_{50}:

30 μM, 15 μM, 7.5 μM, 3.75 μM, 1.875 μM, 0.9375 μM, 0.46875 μM, and 0.23438 μM.

STANDARD DRUG:

Standard drug used in the MTT assay was doxorubicin.

ATCC #: CRL-2522

References:

1. Mannerström, M., Toimela, T., Sarkanen, J., & Heinonen, T. (2017). Human BJ Fibroblasts is an Alternative to Mouse BALB/c 3T3 Cells inIn VitroNeutral Red Uptake Assay. *Basic & Clinical Pharmacology & Toxicology*, *121*, 109-115. doi: 10.1111/bcpt.12790
2. Price, P., & J. McMillan1, T. (1990). Use of the Tetrazolium Assay in Measuring the Response of Human Tumor Cells to Ionizing Radiation. *CANCER RESEARCH*, *50*, 1392-1396. Retrieved from https://cancerres.aacrjournals.org/content/canres/50/5/1392.full.pdf.

C. HeLa cells (Cervical Cancer) assay

Cytotoxic activity of compounds was evaluated in 96-well flat-bottomed micro plates by using the standard MTT (3-[4, 5-dimethylthiazole-2-yl]-2, 5-diphenyl-tetrazolium bromide) colorimetric assay[40]. For this purpose, HeLa cells (Cervical Cancer) were cultured in Minimum Essential Medium Eagle, supplemented with 5% of fetal bovine serum (FBS), 100 IU/ml of penicillin and 100 μg/ml of streptomycin in 75 cm^2 flasks, and kept in 5% CO_2 incubator at 37°C. Exponentially growing cells were harvested, counted with haemocytometer and diluted with a particular medium. Cell culture with the concentration of $6x10^4$ cells/ml was prepared and introduced (100 μL/well) into 96-well plates. After overnight incubation, medium was removed and 200 μL of fresh medium was added with different concentrations of compounds (1-30μM). After 48 hrs, 200 μL MTT (0.5 mg/ml) was added to each well and incubated further for 4 hrs. Subsequently, 100μL of DMSO was added to each well. The extent of MTT reduction to formazan within cells was calculated by measuring the absorbance at 570 nm, using a micro plate reader (Spectra Max plus, Molecular Devices, CA, USA). The cytotoxicity was recorded as concentration causing 50% growth inhibition (IC_{50}) for HeLa. The percent inhibition was calculated by using the following formula:

% inhibition = 100-((mean of O.D of test compound – mean of O.D of negative control)/ (mean of O.D of positive control – mean of O.D of negative control)*100).

The results (% inhibition) were processed by using Soft- Max Pro software (Molecular Device, USA).

STANDARD DRUG:

Standard drug used in the MTT assay was doxorubicin.

Reference:

1. Mosmann T. Rapid colorimetric assay for cellular growth and survival: application to proliferation and cytotoxicity assays. *J Immunol Meth* **1983**, *65*, 55–63.

D. MCF-7 breast cancer cell line assay.

MTT assay is a rapid and quantitative assay capable of measuring the surviving or proliferating cells. It is based on the reduction of tetrazolium salt MTT (3-(4,5-dimethylthiazol-yl)-2,5-diphenyl tetrazolium bromide) by various dehydrogenase enzymes present in living cells. The tetrazolium ring present in MTT is cleaved in active mitochondria, which results in production of purple colored formazan crystals. These crystals are dissolved in DMSO and optical density of the color produced is taken by spectrophotometer (ELISA reader), with high efficiency and accuracy.

This assay was optimized and slightly modified according to our cell lines, i.e. MCF-7 breast cancer cell line.

METHODOLOGY:

1. MCF-7 cell line were cultured in Dulbecco's modified Eagle medium (containing 10% fetal bovine serum) in 75 cc flasks, and kept in 5% CO_2 incubator at 37 ^{o}C.
2. Upon confluency, cells were harvested and plated in 96-well tissue culture treated flat bottom plates (seeding density 8,000 cells/well for MCF-7) in 100 µL medium.
3. Next day, compounds were added in triplicate at 50 µM concentration, and incubated for 48 hours. For natural extracts, the concentration was 50 µg/mL.
4. After 48 hrs incubation, the compounds were removed and 200 µL MTT at 0.5 mg/mL was added to each well and incubated at 37°C for 3 hours.
5. Formazan crystals, formed by reduction of MTT were dissolved in 100 µL DMSO and absorbance was taken at 570 nm using micro-plate reader (Spectra Max plus, Molecular Devices, CA, USA). .
6. The percent inhibition or decrease in viable cells was calculated by following formula:

   % Inhibition = 100 - (mean of O.D. of test compound - mean of O.D. of negative control) / (mean of O.D. of positive control - mean of O.D. of negative control) x 100)

7. If compounds showed 50% of more inhibition, they were further processed for IC_{50} calculation. Twenty (20) mM stock concentration of compounds were diluted to working concentration of 50 µM, and then further serial dilutions were made in order to get less than 50% inhibition. The IC_{50} was then calculated by using EZ-fit5 software.

STANDARD DRUG:

Standard drug used in the MTT assay was doxorubicin.

ATCC # (MCF-7): HTB-22

Reference:

1. Scudiero, D. A.; Shoemaker, R.H.; Paull, K.D.; Monks, A.; Tierney, S.; Nofziger, T.H.; Currens, M.J.; Seniff, D.; Boyd, M.R. Evaluation of a soluble tetrazolium/formazan assay for cell growth and drug sensitivity in culture using human and other tumor cell lines. *Cancer Res.* **1988**, *48*, 4827–4833.
